# Bioproduction, characterization, anticancer and antioxidant activities of extracellular melanin pigment produced by newly isolated microbial cell factories *Streptomyces glaucescens* NEAE-H

**DOI:** 10.1038/srep42129

**Published:** 2017-02-14

**Authors:** Noura El-Ahmady El-Naggar, Sara M. El-Ewasy

**Affiliations:** 1 Department of Bioprocess Development, Genetic Engineering and Biotechnology Research Institute, City for Scientific Research and Technological Applications, Alexandria, Egypt

## Abstract

In this present study, a newly isolated strain, *Streptomyces* sp. NEAE-H, capable of producing high amount of black extracellular melanin pigment on peptone-yeast extract iron agar and identified as *Streptomyces glaucescens* NEAE-H. Plackett–Burman statistical design was conducted for initial screening of 17 independent (assigned) variables for their significances on melanin pigment production by *Streptomyces glaucescens* NEAE-H. The most significant factors affecting melanin production are incubation period, protease-peptone and ferric ammonium citrate. The levels of these significant variables and their interaction effects were optimized by using face-centered central composite design. The maximum melanin production (31.650 μg/0.1 ml) and tyrosinase activity (6089.10 U/ml) were achieved in the central point runs under the conditions of incubation period (6 days), protease-peptone (5 g/L) and ferric ammonium citrate (0.5 g/L). Melanin pigment was recovered by acid-treatment. Higher absorption of the purified melanin pigment was observed in the UV region at 250 nm. It appeared to have defined small spheres by scanning electron microscopy imaging. The maximum melanin yield was 350 mg dry wt/L of production medium. *In vitro* anticancer activity of melanin pigment was assayed against skin cancer cell line using MTT assay. The IC_50_ value was 16.34 ± 1.31 μg/ml for melanin and 8.8 ± 0.5 μg/ml for standard 5-fluorouracil.

Melanins are macromolecules formed by oxidative polymerization of phenolic or indolic compounds. Often the resulting pigments are brown or black in color but many other colors have also been observed. Melanins are also hydrophobic and negatively charged^1^. The biosynthesis of melanin is initiated from L-tyrosine via a series of enzymatic and nonenzymatic reactions by the enzyme tyrosinase^2^. First, tyrosinase (monophenol monooxygenase EC 1.14.18.1) catalyzes oxidation of L-tyrosine to L-3, 4- dihydroxyphenyl alanine (L-DOPA), which is further converted into dopachrome. Dopachrome is converted to melanin by a series of nonenzymatic oxidoreduction reactions^3^. Tyrosinases from different biological sources have been utilized for the synthesis of L-DOPA and the removal of phenolic compounds from wastewaters^4^.

There are three types of melanins i.e. eumelanins, pheomelanins and allomelanins. Eumelanins are black to brown color pigments produced by oxidative polymerization of tyrosine (and/or phenylalanine) to L-DOPA, which is further converted into dopachrome and then to melanin^1^. Eumelanin is the predominant pigment synthesized in humans and microorganisms^5^. Pheomelanins are red or yellow color pigments which are initially synthesized just like eumelanins, but DOPA undergoes cysteinylation (Supplementary Figure S1) (incorporation of cysteine in the polymer)^6^ and contain sulphur. The allomelanins forming the third class are heterogeneous pigments include nitrogen free heterogeneous group of polymers formed from a variety of sources like dihydrofolate, homogentisic acid, catechols, etc.^7^.

Melanins play important roles in microorganisms against thermal, chemical (heavy metal and oxidizing agent) and biochemical stresses (reactive oxygen generated by the exposure of solar UV radiation)^8^. The melanin synthesized by microbes shows metal ions chelating ability^9^. It has also been shown to provide structural rigidity to cell walls and to help to store water and ions^1^. Melanins may also play a role in protecting against antimicrobial drugs^10^ whereas in plants melanin is incorporated in their cell walls as strengtheners^11^. In humans, melanin not only determines the skin color, but also plays an important role in protecting against UV radiation^12^ and its lack leads to several abnormalities and diseases. Due to their chemical composition, melanins have physicochemical properties that allow them to act as ultraviolet absorbers, cation exchangers, drug carriers, amorphous semiconductors, X-ray and γ-ray absorbers^13^. Water soluble melanins are used in sun-screens, solid plastic films, lenses, paints, varnishes, and other surface protection formulations to provide greater UV protections^14^. Melanins have important biological activities, including antimicrobial^15^, antitumor^16^, antivenin activity^17^ and liver protecting activity^18^. There is report of bacterial melanins with anti inflammatory activity^19^. Hoogduijn *et al*.^20^ observed that melanin protects melanocytes and keratinocytes from the induction of DNA strand breaks by hydrogen peroxide, indicating that this pigment has an important antioxidant role in the skin^21^. AIDS treatment reveals the selective antiviral activity of synthetic soluble melanin against human immunodeficiency virus^22^.

Recently, melanin production by microorganisms has attracted attention as an environmentally friendly and economic alternative to chemical production^23^. Actinomycetes are the biotechnologically valuable bacteria which are well exploited for secondary metabolites^24^. Naturally they are able to synthesize and excrete dark soluble pigments, the melanins or melanoid pigments^25^.

The objectives of this study is to isolate and identify efficient extracellular melanin producing *Streptomyces* sp. for pharmaceutical needs, to identify a suitable promotional medium for enhancing melanin production and for characterization of the extracted microbial pigment.

## Results and Discussion

**Figure 1:**
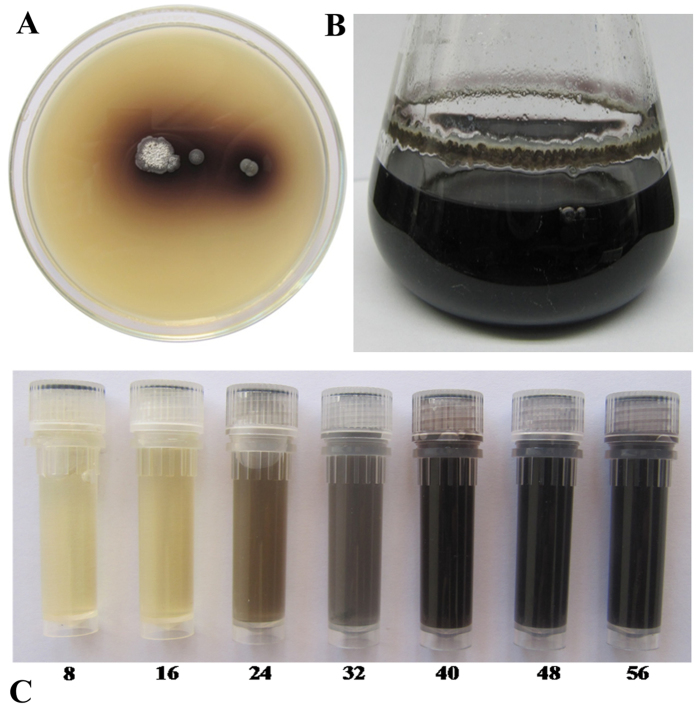
(**A**) Melanin production by *Streptomyces glaucescens* strain NEAE-H in peptone yeast extract iron agar; (**B**) culture broth after 2 days of incubation; (**C**) tubes containing culture samples at different elapsed times. Number below each vial indicates incubation time in hours.

The total of one hundred and thirty morphologically different actinomycete strains were isolated from different localities in Egypt and Saudi Arabia. All these isolates were purified and screened for the extracellular synthesis of melanin on peptone yeast extract iron agar and tyrosine agar using plate method. The formation of brown or black zone around the colonies of the tested isolates on peptone-yeast extract iron and/or tyrosine agar significantly reveals the synthesis of melanin. Melanin production by *Streptomyces* sp. strain NEAE-H in peptone yeast extract iron agar is shown in Fig. 1A. Also the isolates were screened for the extracellular synthesis of melanin in peptone yeast extract iron broth. Melanin production by *Streptomyces* sp. strain NEAE-H in peptone yeast extract iron broth after 2 days of incubation is shown in Fig. 1B and at different elapsed times is shown in Fig. 1C.

Out of the 130 isolates screened, only 9% of the isolates had the ability to produce melanin in the recommended melanin production media. The results correlated with previous findings where streptomycetes from various sources were screened and only less than 10% were found to produce melanin pigments^26^. Only very few actinomycetes from different ecological habitats exhibited the ability to produce melanin. It was noticed that out of 30 isolates from Egyptian soil, only a single strain had the ability to produce melanin^27^. Nine strains among 180 (5%) *Streptomyces* isolates from soil samples of Gulbarga region produced a diffusible dark brown pigment on both peptone-yeast extract iron agar and synthetic tyrosine agar^25^. The most promising isolate, *Streptomyces* sp. strain NEAE-H, was selected as potential isolate for the synthesis of melanin and identified on the basis of morphological, cultural, physiological and chemotaxonomic properties, together with 16 S rRNA sequence.

### Morphology and cultural characteristics of the strain no. NEAE-H

**Figure 2:**
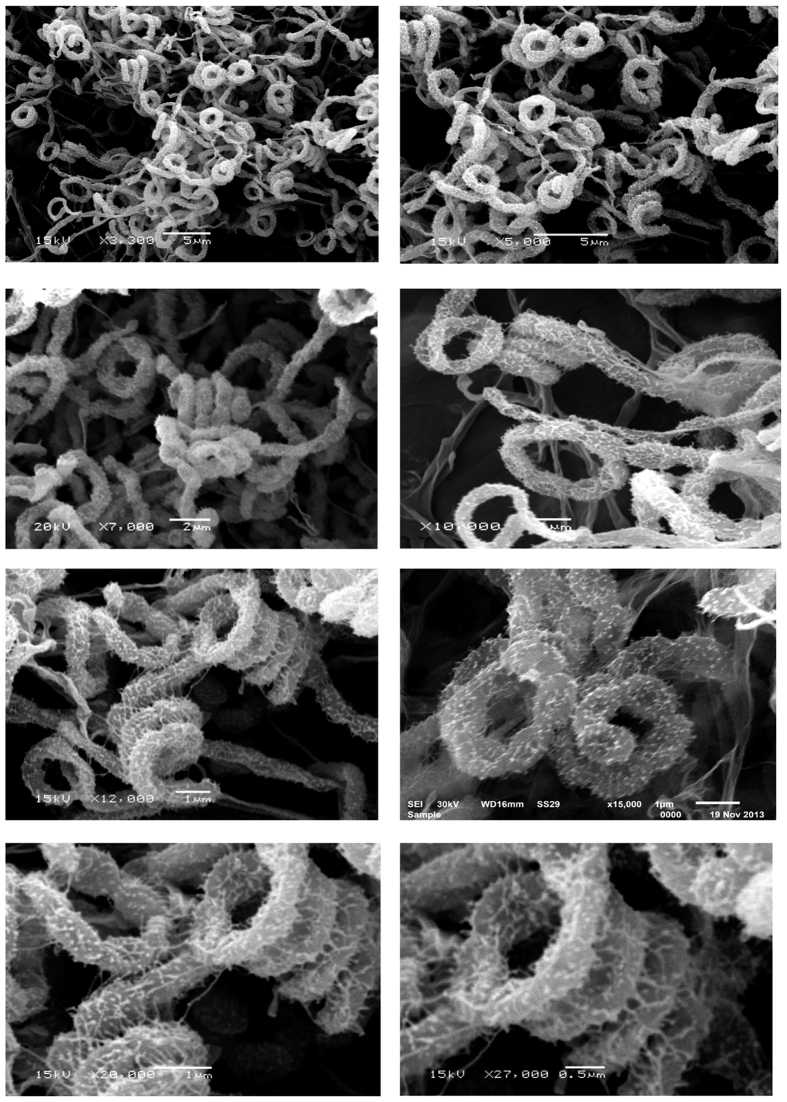
Scanning electron micrograph showing the spore-chain morphology and spore-surface ornamentation of strain NEAE -H grown on starch nitrate agar medium for 14 days at 30 °C at different magnification.

Strain NEAE-H had morphological characteristics that were consistent with members of the genus *Streptomyces*. Strain NEAE-H develops abundant and well-developed substrate and aerial mycelium. It grew well on all tested media (tryptone-yeast extract agar, yeast extract -malt extract agar, oatmeal agar, inorganic salt-starch agar, glycerol–asparagine agar, peptone-yeast extract iron agar and on tyrosine agar) (Supplementary Table S1). The color of the mature sporulating aerial mycelium was green on several standard tested media (Supplementary Figure S2). Reverse side of colony is yellowish brown on tryptone-yeast extract agar; brownish orange on yeast extract -malt extract agar; yellowish green on oatmeal agar and brown on inorganic salts-starch agar, glycerol asparagines agar, peptone-yeast extract iron agar and tyrosine agar; substrate pigment is not pH indicator. Yellow pigments produced in oatmeal agar medium and brown pigments produced in inorganic salts-starch agar, glycerol asparagines agar, peptone-yeast extract iron agar and tyrosine agar media (Supplementary Table S1). Verticils are not present and the mycelium does not fragment. From electron microscopic observations, it was found that strain NEAE-H had spirals-type spore chains, mature spore chains are short. Spore surface is hairy; hairs are coarse, showing some tendency towards spines. Spore shape is globose to oval, this morphology is seen on starch nitrate agar medium (Fig. 2).

### Physiological and chemotaxonomic characteristics

The physiological characteristics of strain NEAE-H are shown in Table 1. Melanin pigments are formed in peptone-yeast-iron agar and tyrosine agar. Lecithinase activity, α–amylase (starch hydrolysis), protease (degradation of casein), cellulase (growth on cellulose), uricase, gelatinase (gelatin liquification) and asparaginase of strain NEAE-H were produced while chitosanase was not produced. Coagulation and peptonization of milk were positive while hydrogen sulphide production and reduction of nitrate to nitrite were negative. The optimal growth temperature was 30 °C and optimal pH was 7.0. The isolate exhibited NaCl tolerance up to 5% (w/v). D-fructose, D-xylose, D-galactose, D-glucose, L-arabinose, ribose, D-mannose, sucrose, maltose, rhamnose and cellulose are utilized as sole carbon sources, while raffinose is weakly utilized as sole carbon source. It exhibited no antimicrobial activities against *Staphylococcus aureus, Alternaria solani, Bipolaris oryzae, Sacchromyces cerevisiae, Candida albicans, Bacillus subtilis, Escherichia coli, Pseudomonas aeruginosa, Rhizoctonia solani, Fusarium oxysporum, Aspergillus niger* and *Klebsiella pneumoniae.* Strain NEAE-H is aerobic, mesophilic and Gram-positive actinomycete.

### 16S rRNA gene sequence comparisons and phylogenetic analysis

**Figure 3:**
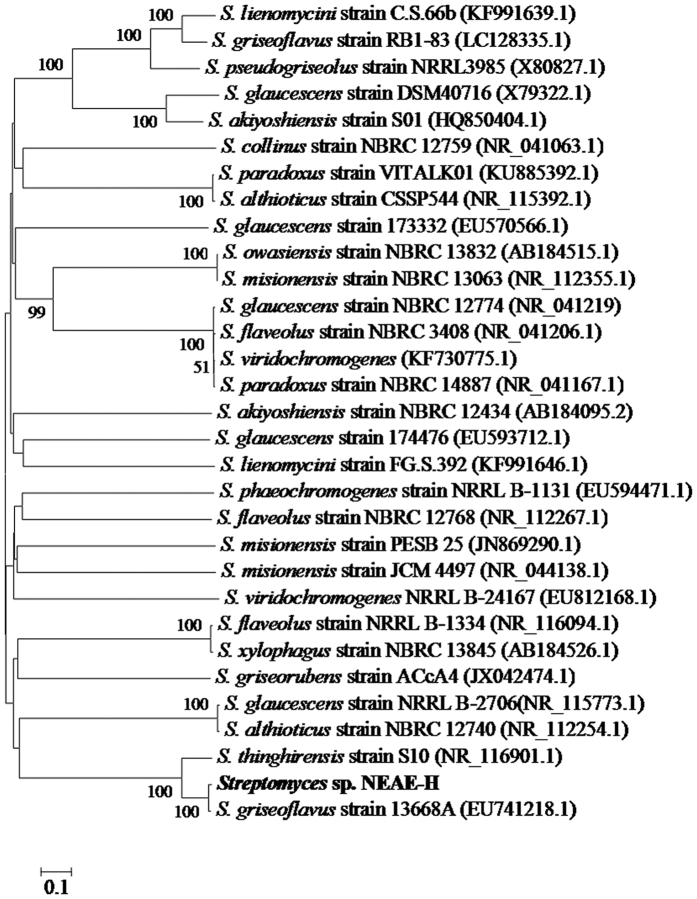
Neighbour-joining phylogenetic tree based on 16 S rRNA gene sequences, showing the relationships between strain NEAE-H and related species of the genus *Streptomyces*. Only bootstrap values above 50%, expressed as percentages of 500 replications, are shown at the branch points. GenBank sequence accession numbers are indicated in parentheses after the strain names. Phylogenetic analyses were conducted in the software package MEGA4. Bar, 0.1 substitution per nucleotide position.

A BLAST search of the GenBank database using 1487 bp 16 S rRNA gene sequence of strain NEAE-H showed its similarity to that of many members of the genus *Streptomyces.* 16 S rRNA gene sequence similarities between the strain NEAE-H and these type strains of the genus *Streptomyces* were between 98 and 100%. A phylogenetic tree (Fig. 3) based on 16 S rRNA gene sequences of members of the genus *Streptomyces* was constructed according to the bootstrap test of neighbor-joining algorithm method of Saitou and Nei^28^ with MEGA4^29^. This tree shows the close phylogenetic association of strain NEAE-H with certain other *Streptomyces* species. Phylogenetic analysis indicated that the strain NEAE-H consistently falls into a clade together with *Streptomyces glaucescens* strain NRRL B-2706 (GenBank/EMBL/DDBJ accession No. NR_115773.1, similarity 100%), *Streptomyces althioticus* strain NBRC 12740 (GenBank/EMBL/DDBJ accession No. NR_112254.1, similarity 99%), *Streptomyces thinghirensis* strain S10 (GenBank/EMBL/DDBJ accession No. NR_116901.1, similarity 99%), *Streptomyces griseoflavus* strain 13668 A (GenBank/EMBL/DDBJ accession No. EU741218.1, similarity 99%).

On the basis of the previously collected data and in view of the comparative study of the morphological, cultural and physiological characteristics of isolate No. NEAE-H in relation to its closest phylogenetic neighbours of the genus *Streptomyces* (Table 1), it is most closely related to the type strain of *Streptomyces glaucescens* strain NRRL B-2706 (GenBank/EMBL/DDBJ accession No. NR_115773.1, the highest degree of similarity 100%)^30^. Therefore, this strain was identified as *Streptomyces glaucescens* strain NEAE-H.

### Evaluation of the factors affecting the extracellular synthesis of melanin using Plackett-Burman design

The production of a diffusible dark brown pigment on complex organic media is so significant that it has long been regarded as a key characteristic for the identification and classification of *Streptomyce*s. The method of testing melanin formation by L-DOPA as substrate is used to confirm whether the diffusible pigments produced are melanoid (dark brown) or merely a brown substance, especially when complex organic media are employed^25^. The experiment was conducted in 20 runs to study the effect of the selected variables on the production of melanin. Plackett-Burman experiments showed a markedly wide variation of melanin production from 5.72 to 17.96 μg/0.1 ml of medium (Table 2); this variation reflected the importance of medium optimization to attain higher melanin production. The maximum melanin production (17.96 μg/0.1 ml of medium) and tyrosinase activity (5454.27  U/ml) were achieved in the run number 17, while the minimum melanin production (5.72 μg/0.1 ml of medium) and tyrosinase activity (996.05 U/ml) were observed in the run number 8 (Table 2).

The relationship between a set of independent variables and melanin production is determined by a mathematical model called multiple-regression model. The data revealed that, medium volume (E) and potassium nitrate (J) are insignificant variables with zero effect (0.0) and zero percent of contribution (0.0). Lower % of contribution indicated higher *p*-value. Thus instead of starting with the maximum model effects, backward regression at alpha 0.15 was applied to eliminate the effect of medium volume and potassium nitrate. Then, the model fitted for the test of significance. Statistical analysis of the response was performed which is represented in Table 3, Supplementary Table S2.

**Figure 4:**
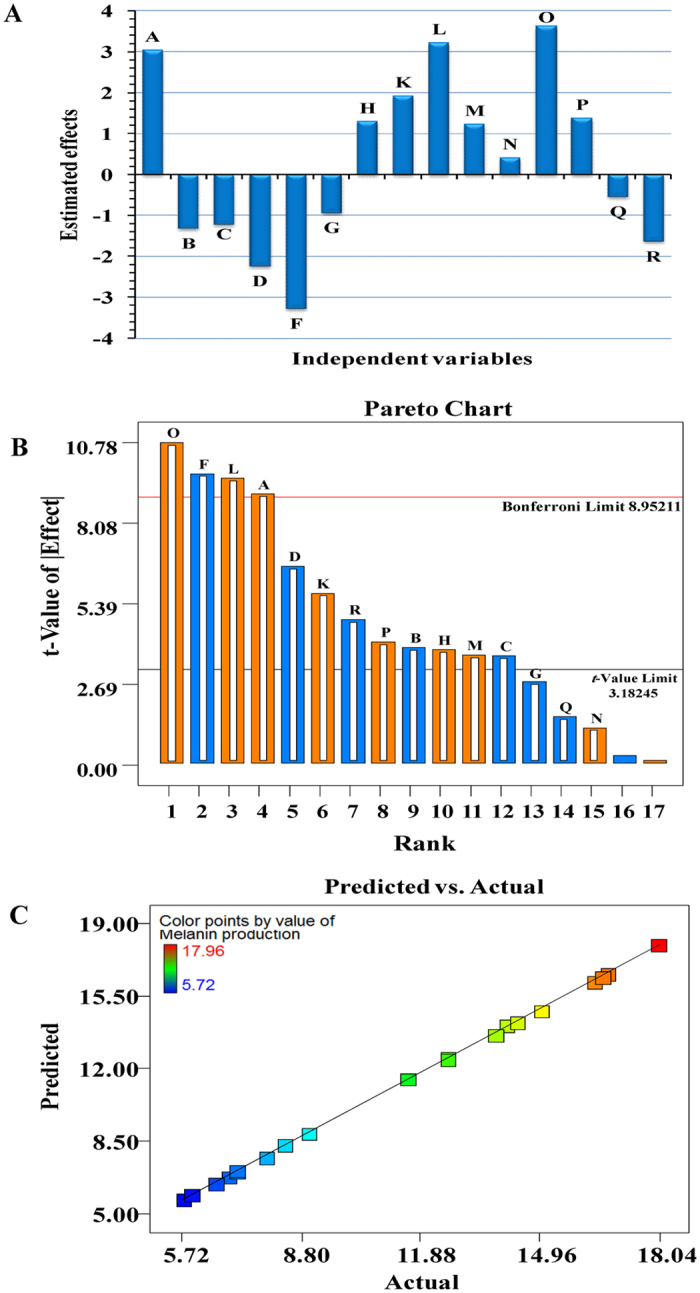
(**A**) The main effects of the factors affecting melanin production, eight variables affect positively melanin production, where seven variables affect negatively melanin production, (**B**) The Pareto chart shows the amount of influence of each factor on melanin production, **C)** Correlation between the experimented and predicted values for melanin production by *Streptomyces glau*cescens strain NEAE-H according to the Plackett–Burman experimental results.

Supplementary Table S2 and Fig. 4A show the main effect of each variable on melanin production. With respect to the main effect of each variables, we can see that eight variables from the seventeen different independent variable named incubation period, L-tyrosine, peptone, protease-peptone, yeast extract, K_2_HPO_4_, ferric ammonium citrate and sodium thiosulfate affect positively melanin production, where the seven variables named glycerol, MgSO_4_, NaCl, pH, temperature, agitation speed and starch affect negatively melanin production. The significant variables with positive effect were fixed at high level and the variables which exerted a negative effect on melanin production were maintained at low level for further optimization by a face-centered central composite design. Glycerol, KNO_3_, MgSO_4_, NaCl and starch were excluded from the production medium due to the low levels of these factors is “0”. Medium volume was maintained at low level for further optimization.

The Pareto chart illustrates the order of significance of the variables affecting melanin production in Plackett-Burman experimental design (Fig. 4B). It displays the absolute values of the effects, and draws a reference line on the chart. Any effect that extends past this reference line is potentially important. Pareto chart in design expert version 7.0 reproduce the relation between *t*-value (effect) vs. ranks. Among the tested variables, ferric ammonium citrate showed the highest positive effect by 13.282%. Next to ferric ammonium citrate, protease-peptone showed positive effect by 11.782%, then incubation period by 11.160% (Supplementary Table S2). Starch showed the highest negative significance by 11.965%. Also, predicted versus actual melanin production plot indicated that, there is a close agreement between the experimental results and theoretical values predicted by the model equation as shown in Fig. 4C, which confirms the adequacy of the model.

The value of the determination coefficient (R^2^ = 0.9998) indicates that 99.98% of the variability in the response was attributed to the given independent variables and only 0.02% of the total variations are not explained by the independent variables. In addition, the value of the adjusted determination coefficient (Adj. R^2^ = 0.9979) is also very high which indicates a high significance of the model^31^. The “Pred R-Squared” of 0.9778 is in reasonable agreement with the “Adj R-Squared” of 0.9979.This indicated a good adjustment between the observed and predicted values. “Adeq Precision” measures the signal to noise ratio. A ratio greater than 4 is desirable. Our ratio of 67.877 indicates an adequate signal (Table 3).

The analysis of variance (ANOVA) of the experimental design was calculated, and the sum of square, mean square, *F*-value, *P*-value and confidence level are given in Table 3. The significance of each coefficient was determined by *p*-values, which are listed in Table 3. The Model *F*-value of 529.93 implies the model is significant. Values of “Prob > *F*” (*P*-value) less than 0.05 indicate model terms are significant. In this case A, B, C, D, F, G, H, K, L, M, N, O, P, Q and R are significant model terms. The analysis showed that, ferric ammonium citrate (O) with a probability value of 0.0006 was determined to be the most significant factor affecting melanin production by *Streptomyces glaucescens* strain NEAE-H at 99.940% confidence followed by protease-peptone (L) (*P*-value = 0.0007) at 99.930% confidence, and starch (F) (*P*-value = 0.0007) at 99.930% confidence, then incubation period (A) (*P*-value = 0.0008) at 99.920% confidence.

The coefficient of variation % (C.V.%) is a measure of residual variation of the data relative to the size of the mean. Here a lower value of C.V. (1.690%) indicates a greater reliability of the experimental performance. The predicted residual sum of squares (PRESS) statistic is used as an indication of the predictive power of a model. A model with a small value of PRESS statistic indicates better prediction. Our value of PRESS is 7.29. The model shows standard deviation and mean value of 0.191and 11.294, respectively.

By neglecting the terms that were insignificant (*P* > 0.05) (Supplementary Table S2), the first order polynomial equation was derived representing melanin production as a function of the independent variables:


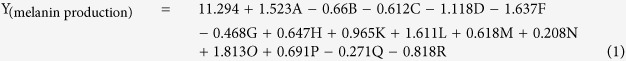


Where Y is the response (melanin production) and A, B, C, D, F, G, H, K, L, M, N, O, P, Q, R are incubation period, pH, temperature, agitation speed, starch, glycerol, L-tyrosine, peptone, protease-peptone, yeast extract, K_2_HPO_4_, ferric ammonium citrate, sodium thiosulfate, MgSO_4_ and NaCl.

In a confirmatory experiment, to evaluate the accuracy of Plackett-Burman, a medium, which expected to be optimum of the following composition:(g/L: L-tyrosine 3, peptone 15, protease-peptone 4, yeast extract 1, K_2_HPO_4_ 1, ferric ammonium citrate 0.4, sodium thiosulfate 0.08), incubation period 5 days, pH 7, temperature 30 °C, agitation speed 100 rpm and medium volume (50 ml/250 ml flask) gives 19.17 μg melanin/0.1 ml of medium which is higher than result obtained from the basal medium before applying Plackett-Burman by about 2.24 times (8.57 μg/0.1 ml of medium).

### Optimization by face-centered central composite design

The face-centered central composite design was employed to study the interactions among the significant variables and also determine their optimal levels. Results of Placket-Burman design revealed that incubation period, protease-peptone and ferric ammonium citrate were the most significant positive independent variables affecting melanin production, thus they were selected for further optimization using face-centered central composite design.

In this study, a total of 20 experiments with different combination of incubation period (X_1_), protease-peptone (X_2_) and ferric ammonium citrate (X_3_) were performed and the results of experiments are presented along with predicted response and residuals in Table 4. Concentrations of three independent variables at different coded and actual levels of the variables also presented in Table 4. The central point was repeated six times (run order: 1, 3, 10, 13, 15 and 20). The maximum melanin production (31.650 μg/0.1 ml of medium) and tyrosinase activity (6089.10U/ml) were achieved in central point runs number 1, 3, 10, 13, 15 and 20 under the conditions of incubation period (6 days), protease-peptone (5 g/L) and ferric ammonium citrate (0.5 g/L), while the minimum melanin production (3.006 μg/0.1 ml of medium) and tyrosinase activity (2174.51 U/ml) was observed in run number 17 under the conditions of incubation period (8 days), protease-peptone (3 g/L) and ferric ammonium citrate (0.3 g/L) (Table 4).

### Multiple regression analysis and ANOVA

The data were analyzed using Design Expert^®^ 7.0 for Windows to perform statistical analysis. The positive coefficients for X_2_, X_3_, X_1_ X_2_, X_1_ X_3_, X_2_ X_3_ (Table 5) indicate that linear effect of X_2_, X_3_ and interaction effects for X_1_ X_2_, X_1_ X_3_, X_2_ X_3_ increase melanin production, while other negative coefficients indicate decrease in melanin production.

The adequacy of the model was checked using analysis of variance (ANOVA) which was tested using Fisher’s statistical analysis and the results are shown in Table 5. The Model *F*-value of 66.903 with a very low probability value (*P* model > *F* 0.0001) implies the model is significant. It can be seen from the degree of significance that the quadratic effect of incubation period (X_1_), protease-peptone (X_2_) and ferric ammonium citrate (X_3_) are significant model terms (*P* value > 0.05). Linear coefficients and interaction between three variables are not significant (*P* value > 0.05) indicating that there is no significant correlation between three variables and that they did not help much in increasing the production of melanin (Table 5).

The determination coefficient (R^2^) of the model was 0.9837 (Table 5). Therefore, the present R^2^-value reflected a very good fit between the observed and predicted responses, and implied that the model is reliable for melanin production in the present study. The “Pred R-Squared” of 0.9025 is in reasonable agreement with the “Adj R-Squared” of 0.9690. This indicated a good adjustment between the observed and predicted values. “Adeq Precision” ratio of 19.2714 indicates an adequate signal to noise ratio. A lower value of C.V. (11.715) indicated a better precision and reliability of the experimental performance^32^. Value of PRESS is 257.139. The model shows standard deviation and mean value of 2.076 and 17.720, respectively (Table 5).

In order to evaluate the relationship between dependent and independent variables and to determine the maximum melanin production corresponding to the optimum levels of incubation period (X_1_), protease-peptone (X_2_) and ferric ammonium citrate (X_3_), a second-order polynomial model (Equation 2) was proposed to calculate the optimum levels of these variables and defines predicted response (Y) in terms of the independent variables (X_1_, X_2_ and X_3_):


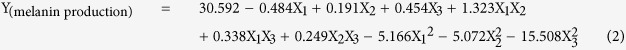


Where Y is the response (melanin production) and X_1_, X_2_ and X_3_ are incubation period, protease-peptone and ferric ammonium citrate, respectively.

The fit summary results are presented in Supplementary Table S3. The aim of sequential model sum of squares is to select the highest order polynomial where terms are significant; quadratic model type was selected to be the proper model that fit the FCCD of melanin production by *Streptomyces glaucescens* strain NEAE-H, where fit summary results showed that, the quadratic model is a highly significant model with a very low probability value [(*P*_model_ > *F*) < 0.0001]. The model summary statistics focus on the models that have lower standard deviation and higher adjusted and predicted R-squared; the model summary statistics of the quadratic model showed the smallest standard deviation of 2.076 and the largest adjusted and predicted R-squared of 0.9690 and 0.9025 respectively.

### Three dimensional plots

**Figure 5:**
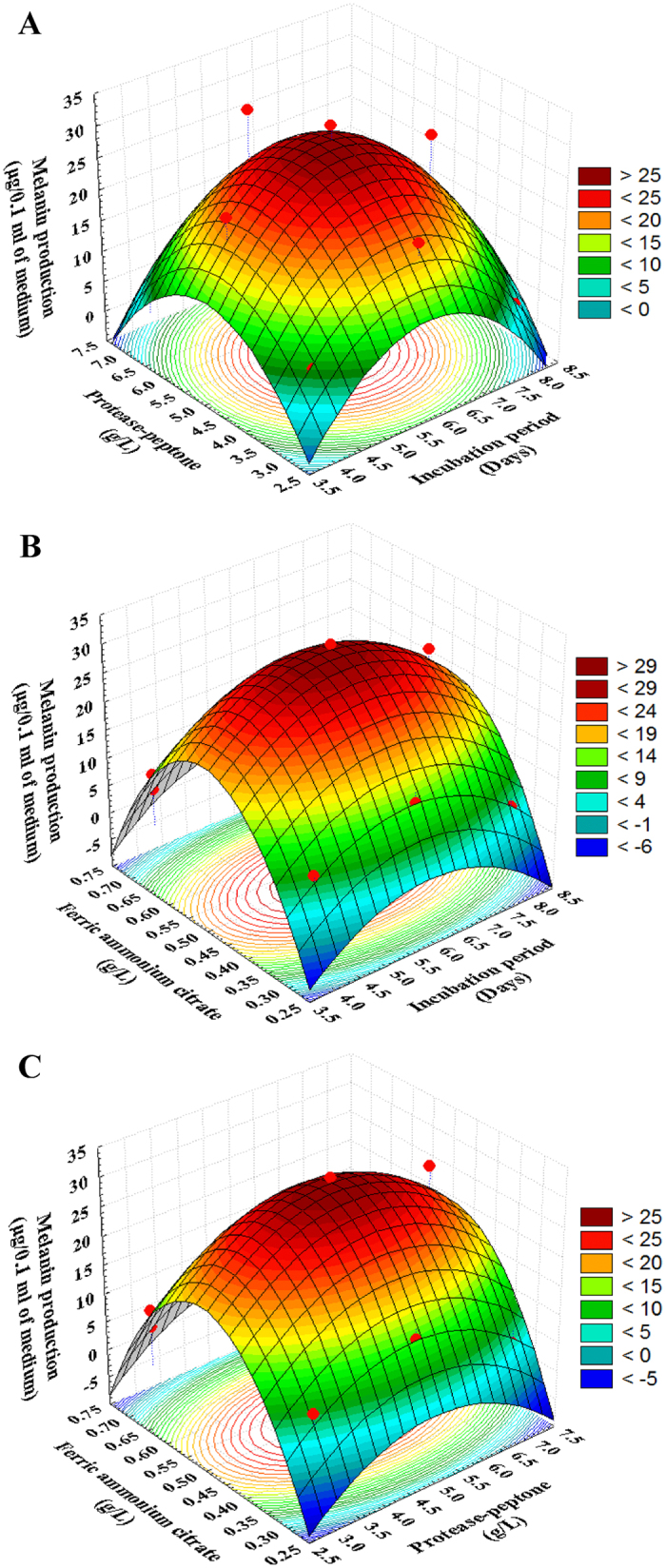
Three-dimensional response surface plots for melanin production showing the interactive effects of incubation period (X_1_), protease-peptone (X_2_) and ferric ammonium citrate (X_3_) when one of the variables is fixed at optimum value and the other two are allowed to vary.

The three dimensional response surface curves were plotted by statistically significant model to understand the interaction of the variables and the optimal levels of each variable required for the optimal melanin production. Three dimensional plots for the significant pair-wise combinations of the three variables (X_1_ X_2_, X_1_ X_3,_ and X_2_ X_3_) were generated by plotting the response (melanin production) on Z-axis against two independent variables while keeping the other variable at its center point (zero levels) (shown in Fig. 5A–C). Figure 5A represents the three dimensional plot as function of incubation period (X_1_), protease-peptone (X_2_) on the production of melanin. Maximum melanin production was clearly situated close to the central point of the incubation period and protease-peptone. Further increase or decrease led to the decrease in the production of melanin.

The maximum pigment production was observed on 6^th^ day of incubation. This result was in agreement with the finding of Rani *et al*.^33^ who extracted the highest amount of crude melanin at 6^th^ day from halophilic black yeast *Hortaea werneckii.* In contrast, Amal *et al*.^34^ and Vasanthabharathi *et al*.^35^ reported that the maximum level of pigment formation by *Streptomyces* was observed on 10^th^ and 7^th^ day of incubation period respectively after which it slowly declined. The maximum amount of melanin pigments was synthesized by the fungus *Aspergillus carbonicus* at 15^th^–25^th^ day’s incubation period^36^.

Quadri and Agsar^37^ reported that simple nitrogen source tyrosine gave the maximum production of melanin by thermo-alkaliphilic *Streptomyces* followed by phenylalanine. Tyrosine has given the maximum production when compared to complex nitrogen sources. Potassium nitrate was reported as the best nitrogen source for the experimental actinomycete isolate to produce melanin^27^. The nitrogen source utilized varies among different species of *Streptomyce*s. The formation of brown color for *Streptomyces* isolates on peptone-yeast extract iron agar was observed by Vasanthabharathi *et al*.^35^. Twenty-one cultures produced a diffusible dark brown pigment on peptone-yeast extract-iron-agar, but failed to do so on synthetic tyrosine-agar. In these cases, the growth or the production of the enzyme is not enough to be detected on synthetic tyrosine-agar^38^. Proteose peptone is enzymatic digests of protein. It is rich in peptides with the higher molecular weight.

Figure 5B depicts the incubation period (X_1_) and ferric ammonium citrate (X_3_) interactions. At moderate levels of incubation period and ferric ammonium citrate, the production of melanin was high. The graph pointed a decline in production level when the interaction was carried beyond high and low levels of incubation period and ferric ammonium citrate. The organism reduced ferric citrate by ferric reductase activity, converting Fe^3+^ to Fe^2+^ which might facilitate iron acquisition/assimilation by providing a ferrous iron source to growing *Streptomyces*. Increases in the levels of ferric reductase activity in culture supernatants of *Legionella pneumophila* correlated with increased pigmentation. *Legionella pneumophila* is one of only a small number of microorganisms in which melanin secretion has been linked to ferric reduction^39^. A study reports that iron levels can modulate the transcriptional control of melanin biosynthesis in *C. neoformans*^40^. Fe^2+^ ion is required as cofactor for the activity of several aromatic hydroxylases, such as phenylalanine, tryptophan and tyrosine hydroxylases^41^. With regard to a possible mechanism for the observed effect of iron, that the expression of the hydroxylase activity of tyrosinase is dependent on a pre-reduction site of the enzyme^42^.

Figure 5C plot reveals that lower and higher levels of the protease-peptone (X_2_) and ferric ammonium citrate (X_3_) support relatively low levels of melanin production. On the other hand, the maximum melanin production clearly situated close to the central point of the protease-peptone and ferric ammonium citrate. In addition, the interaction terms between these variables were not significant, indicating that there is no significant correlation between each two variables and that they did not help much in increasing the production of melanin.

### Model verification

In order to determine the accuracy of the model and to verify the result, an experiment under the new conditions which obtained from face-centered central composite design was preformed. The predicted optimal levels of the process variables for melanin production by *Streptomyces glaucescens* strain NEAE-H were incubation period (6 days), protease-peptone (5 g/L) and ferric ammonium citrate (0.5 g/L). Melanin production by *Streptomyces glaucescens* strain NEAE-H (31.650 μg/0.1 ml of medium) obtained from the experiment was very close to the response (30.607 μg/0.1 ml of medium) predicted by the regression model, which proved the validity of the model. The verification revealed a high degree of accuracy of the model of 96.70%, indicating the model validation under the tested conditions.

### Extraction of melanin

**Figure 6:**
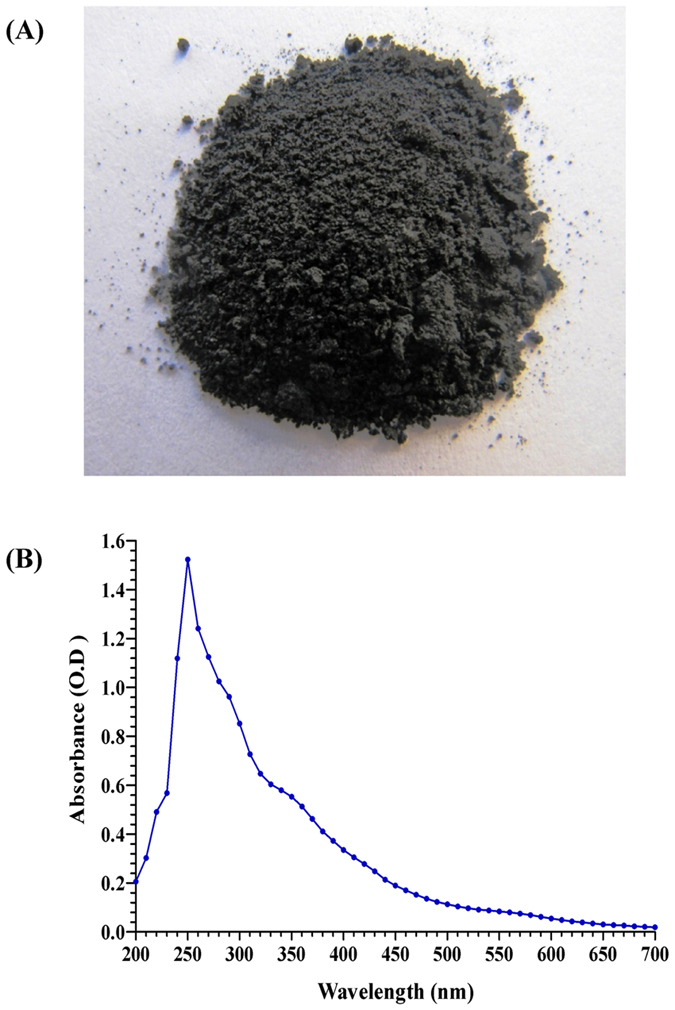
(**A**) Granules of extracted lyophilized melanin; (**B**) UV-visible absorbance spectrum (200–700 nm) of the purified melanin pigment of *Streptomyces glaucescens* strain NEAE-H.

It was suggested that melanin polymers constitute the building blocks of melanin granules^43^. The process of granules formation and their dimension are strongly pH dependent, where a low pH promotes the aggregate growth and a high pH induces the breakup of the granules to small particles-oligomers with a lower degree of polymerization. This process is a consequence of the polyelectrolyte nature of melanin, and it is dependent on the ionization state of melanin groups like carboxylic, phenolic, and aminic groups as well as on the ionic strength of the environment. The physical appearance of the purified melanin is shown in Fig. 6A with a true black color typical of melanins in general.

In the present study, the maximum yield of melanin per liter of peptone yeast extract iron broth was 350 mg dry wt/L of production medium, which is comparable with that of the maximum yield of M8 melanin extracted from peptone yeast extract iron broth culture of *S. bikiniensis* which was 166 mg/L^44^; yeast melanin synthesized by *Yarrowia lipolytica* (160 mg/L)^45^ and tyrosine-mediated melanin production (130 mg/L) by *Klebsiella* sp. GSK^46^.

### UV–visible spectrophotometeric analysis of the purified melanin pigment

The UV-visible absorbance spectrum (200–700 nm) of the purified melanin is shown in Fig. 6B. Higher absorption was observed in the UV region at 250 nm which then decreased towards the visible region, which is the characteristic property of melanin. The absorption peak at 250 nm was similar to the absorption peak for melanin pigment extracted from *Phyllosticta capitalensis*^47^. The melanins of different sources had various maximum UV-Vis absorption peaks, such as the purified *Chroogomphus rutilus* melanin which had maximum absorption peak at 212 nm^48^ and *Actinoalloteichus* sp. MA-32 melanin at 300 nm^15^. Whereas, wavelength scan of melanin synthesized by *Streptomyces bikiniensis* M8 exhibited an absorbance in the UV region with highest absorption peak at 230 nm, but decreased towards the visible region due to the presence of the very complex conjugated structure, which is the characteristic property of melanin^44^. The purified *Chroogomphus rutilus* melanin did not contain nucleic acid or protein because of no obvious absorption peak between 260–280 nm in the UV spectra^48^.

### FTIR analysis of melanin

**Figure 7:**
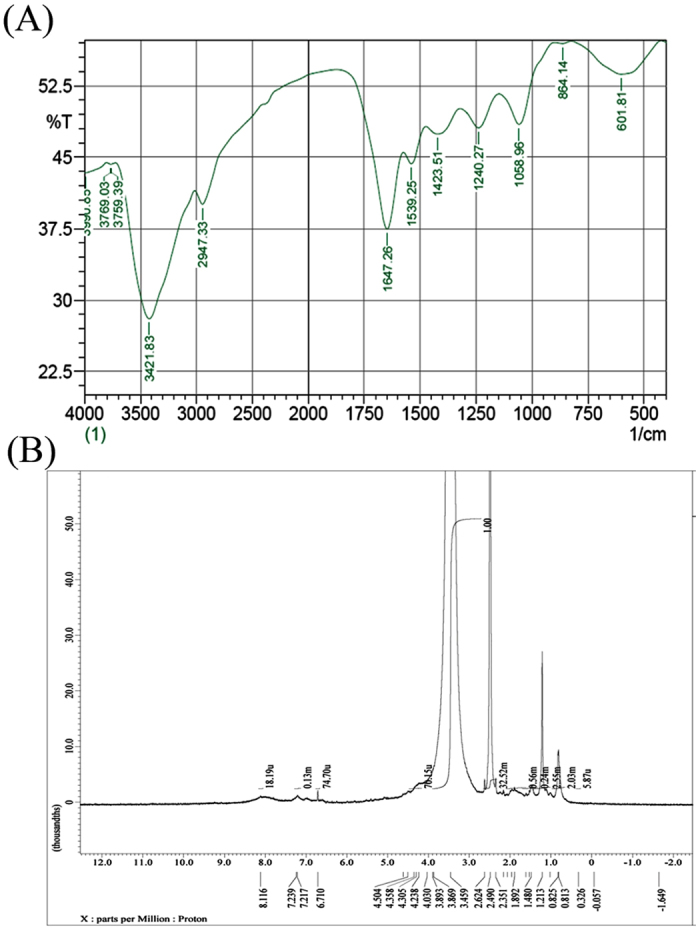
(**A**) FT-IR Spectroscopic analysis of the extracted melanin pigment; (**B**) NMR spectrum of the extracted melanin pigment.

One of the main tests for identifying melanin is the FTIR spectrum. The FTIR spectrum of the extracted melanin (Fig. 7A) shows a peak around 3421.83 cm^−1^, correspond to the OH group, small band at 2947.33 cm^−1^ can be assigned to stretching vibration of aliphatic C-H group^49^. The signals in the 3600–2800 cm^−1^ area are attributed to the stretching vibrations (O-H and N-H) of the amine, amide, or carboxylic acid, phenolic and aromatic amino functions present in the indolic and pyrrolic systems^50^. Peak observed around 1647.26 cm^−1^ is attributed to bending of secondary NH group. The characteristic strong band at between 1650–1620 cm^−1^(1647.26 cm^−1^) attributed to vibrations of aromatic ring C = C of amide I C = O and/or of COO- groups. The N-H bending vibration peak at 1539.25 cm^−1^, indicates that the pigment had typical indole structure of melanin. Bands at ~1400 to 1500 cm^−1^ can be due to aliphatic C-H groups in the melanin pigment^46^. The peak centered at 1423.51 cm^−1^ (CH_2_-CH_3_ bending) characteristic of melanin pigment. Phenolic COH stretching at 1240.27 cm^−1^ relates to phenolic compounds. It was proposed that peaks at 1243 to 1305 cm^−1^ relates to the anhydride group (C-O) in synthetic melanin and all extracted microbial pigments^51^. The peak centered at 1058.96 cm^−1^ is the indication of CH in-plane of aliphatic structure characteristic of melanin pigment. The peak observed at 864.14 cm^−1^ due to aromatics C–H group. Weak bands below 700 cm^−1^ ascribed to alkene C-H substitution in the melanin pigment^46^. The spectroscopic properties of the pigment extracted from *Streptomyces glaucescens* strain NEAE-H correlated with those of melanin produced by various microorganisms as reported previously^46^. On the basis of the above results, it was concluded that the pigment was eumelanin.

### NMR spectrum

NMR spectrum of the purified melanin pigment synthesized by *Streptomyces glaucescens* strain NEAE-H (Fig. 7B) shows resonances between 8.0 and 6.0 ppm and between 0.813 and 4.504 ppm. Resonances between 8.0 and 6.0 ppm in NMR spectra are assigned to aromatic functionality, between 3.2 and about 4.3 ppm are assigned to protons attached to N and/or O, based upon similar assignments in human hair and Sepia melanin^52^.The four broad aromatic resonances at 7.60, 7.35, 7.00, and 6.60 ppm are assigned to indole and/or pyrrole repeat units of the melanin polymer^52^. Resonances between 1.0 and 3.2 ppm are assigned to residual protein.

### Scanning electron micrograph (SEM)

**Figure 8:**
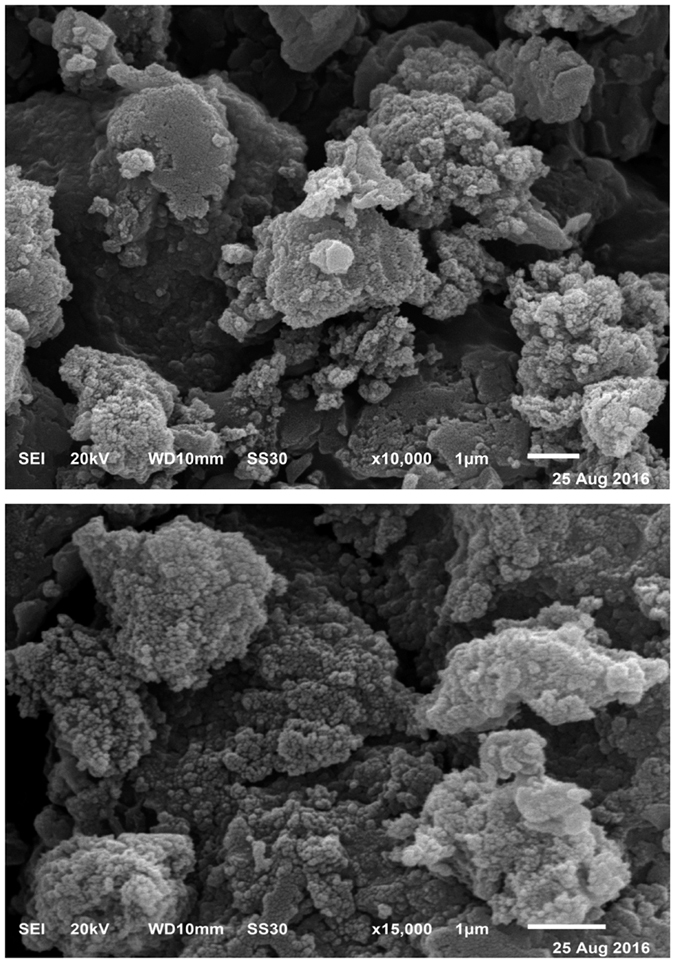
Scanning electron microscope micrographs of the extracted melanin pigment granules at different magnifications showing small spheres.

SEM was used to examine the structure of melanin and the natural melanin appears to be small spheres^5^. In the present study, the purified melanin pigment synthesized by *Streptomyces glaucescens* strain NEAE-H appears to have defined small spheres by SEM imaging (Fig. 8). The natural Sepia melanin sample has a significant structural order with subunits that have a lateral dimension of ~15 nm^53^. Structural order is lacking in the case of melanin produced by *S. bikiniensi*s.

### *In vitro* anticancer activity

The safety pattern of the purified melanin pigment of *Streptomyces glaucescens* strain NEAE-H was assayed on human lung fibroblast (WI-38) and human amnion (WISH). The results revealed that, the treatment IC_50_ on all cells ranged from 37.05 ± 2.40 to 48.07 ± 2.76 μg/ml (Table 6). The anti-proliferative activity of the purified melanin pigment of *Streptomyces glaucescens* strain NEAE-H was assayed for its anticancer activity *in vitro* against skin cancer cell line (HFB4) using MTT assay. The obtained results were expressed as growth inhibitory concentration (IC_50_) values, which represent the melanin pigment concentration required to produce a 50% inhibition of cell growth after 24 h of incubation, compared to untreated controls (Table 6). MTT assay revealed that melanin pigment produced by *Streptomyces glaucescens* strain NEAE-H showed potent cytotoxic activity against HFB4 skin cancer cell line. After 24 h, the total mortality was 81.3% in the highest concentration (100 μg/ml) of melanin comparable to standard 5-fluorouracil which showing 92.2% mortality (Table 6). The IC_50_ value was 16.34 ± 1.31 μg/ml for melanin and 8.8 ± 0.5 μg/ml for standard 5-fluorouracil. From the obtained results, it was obvious that the melanin pigment displayed strong anticancer activity against the tested cell line. Melanin at 50 μg/ml inhibited cell viability by 70.9%.

It can be observed that the purified melanin pigment of *Streptomyces glaucescens* strain NEAE-H showed less cytotoxicity even at high concentrations with an IC_50_ value 37.05 ± 2.40 and 48.07 ± 2.76 against normal non-cancerous, human lung fibroblast and human amnion cells; respectively as compared with standard 5-fluorouracil which showing IC_50_ value 6.68 ± 0.57 and 5.07 ± 0.38; respectively. The potent cytotoxic activity of melanin against HFB4 skin cancer cell line and low cytotoxicity of against normal non-cancerous cells shows that melanin pigment can be used as potential natural anticancer. Arun *et al*.^54^ reported that the *in vitro* inhibition of cell proliferation in HEP 2 carcinoma cell line was concentration dependent. Melanin at 60 μg inhibited the cell viability by 53%. On the other hand, Kurian *et al*.^19^ found that MTT assay revealed that BTCZ31 melanin inhibited growth of L929 cell line, cytotoxic concentration of melanin was found to be 105.4 μg/ml (IC_50_).

### ABTS^+^ radical scavenging (antioxidant) activity

The ability of the melanin pigment to scavenge free radicals was evaluated by inhibition of the oxidation of 2, 2’-azino-bis (3-ethylbenzothiazoline-6-sulfonic acid) (ABTS). The results showed that the purified melanin pigment of *Streptomyces glaucescens* strain NEAE-H showed good antioxidant activity. The results revealed that 100 μg/ml melanin exhibited percentage inhibition 57.2% radical scavenging activity, which was comparable to that of standard antioxidant ascorbic acid showing an activity of 89.6%. ABTS radical was quickly and effectively scavenged by the melanin pigment. Interaction of melanin pigment (antioxidant) with ABTS^+^ transfers hydrogen atoms to ABTS^+^ thus neutralizing its free radical character. Melanin is a polymer able to donate or accept an electron. Melanin pigment interacts with free radicals and other reactive species readily due to the presence of unpaired electrons in its molecules and acts as an antioxidant, suggesting its use as a raw cosmetic material to minimize toxin-induced tissue destruction. Melanin interacts with free radicals via the simple one electron transfer processes^55^.

### Anti-haemolytic activity

*In vitro* anti-haemolytic assay using spectroscopic method was used to evaluate the effect of melanin pigment on the erythrocytes. The results revealed that, melanin pigment exhibited considerable anti-hemolytic activity. Melanin exhibited percentage erythrocyte hemolysis of 11.9%, which was comparable to that of standard ascorbic acid showing percentage erythrocyte hemolysis of 4.4%. The effective anti-hemolytic activity of melanin pigment is because of the ability of phenolic compounds in neutralizing the free radicals and thereby protecting the erythrocytes membrane from destruction and lysis.

## Methods

### Microorganisms and cultural conditions

Streptomyces spp. used in this study were isolated from various soil samples collected from different localities of Egypt and Saudi Arabia. Using standard dilution plate method on Petri plates containing starch nitrate agar medium of the following composition (g/L): Starch, 20; KNO_3_, 2; K_2_HPO_4_, 1; MgSO_4_.7H_2_O, 0.5; NaCl, 0.5; CaCO_3_, 3; FeSO_4_.7H_2_O, 0.01; agar, 20 and distilled water up to 1 L; then plates were incubated for a period of 7 days at 30 ^°^C. Actinomycetes isolates were maintained as spore suspension in 20% (v/v) glycerol at −20 °C for subsequent investigation.

### Screening for melanin producers

During the primary screening, isolates were spot inoculated on peptone yeast extract iron agar (ISP medium 6) plates containing g/L: peptone 15; protease peptone 5; ferric ammonium citrate 0.5; K_2_HPO_4_ 1; sodium thiosulfate 0.08; yeast extract 1 and distilled water 1 L; agar 20 g; pH 7–7.2 and tyrosine agar (ISP medium 7) plates containing g/L: glycerol 15.0; L-tyrosine 0.5; L-asparagine 0.5; K_2_HPO_4_ 0.5; MgSO_4_.7H_2_O 0.5; FeSO_4_.7H_2_O 0.01 and distilled water 1 L; agar 20 g; pH 7.2. Loopful of spores were inoculated on to the agar plates and incubated at 30 °C for 2–6 days. Brown to back zone of diffusible pigment around the colonies in the medium was scored as positive. Melanin production was quantitatively analyzed by seeding the isolates selected after the primary screening into the melanin production medium (peptone yeast extract iron broth). Melanin pigment production was evaluated by measuring O.D. of the filtrate spectrophotometrically at 280 nm.

### Production conditions

100 ml of fermentation medium (peptone yeast extract iron broth) were dispensed in 250 ml Erlenmeyer conical flasks, inoculated with six disks of 9 mm diameter taken from the 7 days old stock culture grown starch nitrate agar medium. The inoculated flasks were incubated for 3–6 days on a rotatory incubator shaker at 100–200 rpm and 30–37 °C. After incubation time, *Streptomyces* cells were collected by centrifugation at 10000 *g* for 10 min. The cell free supernatant was used for assay of tyrosinase activity and melanin formation.

### Assay of tyrosinase activity

The tyrosinase activity test was done to confirm whether the diffusible black/brown pigment formed in peptone yeast extract iron agar and synthetic tyrosine agar is melanin (not melanoid pigments). Tyrosinase activity was assayed as described by the modified method of Robb^56^ by using L-DOPA as a substrate, measuring conversion of L-DOPA to red colored oxidation product dopachrome. 0.5 ml of the enzyme solution was added to freshly prepared 2 ml of 0.1 M potassium phosphate buffer (pH 7) containing L-3, 4- dihydroxyphenyl alanine (L-DOPA, 4 mg/ml of phosphate buffer) as substrate. The reaction mixture was incubated at 37 °C for 15 min. Red coloration resulting from dopachrome formation was monitored by measuring the absorbance spectrophotometrically at 480 nm. One unit of tyrosinase activity was defined as the amount of enzyme that catalyzes the formation of 1 μmol dopachrome per minute at 37 °C.

### Morphology and cultural characteristics

Detailed information is reported in the Supplementary Information.

### Physiological characteristics

Detailed information is reported in the Supplementary Information.

### 16 S rRNA sequencing, sequence alignment and phylogenetic analysis

The preparation of genomic DNA of the strain was conducted in accordance with the methods described by Sambrook *et al*.^57^. The PCR amplification reaction was performed in accordance with the methods described by El-Naggar *et al*.^58^. Sequencing product was deposited in the GenBank database under accession number KJ467537.

The partial 16 S rRNA gene sequence of strain NEAE-H was aligned with the corresponding 16 S rRNA sequences of the type strains of representative members of the genus *Streptomyces* retrieved from the GenBank, EMBL, DDBJ and PDB databases by using BLAST program (https://blast.ncbi.nlm.nih.gov/Blast.cgi?PAGE_TYPE=BlastSearch)^59^ and the software package MEGA4 version 2.1^29^ was used for multiple alignment and phylogenetic analysis. The phylogenetic tree was constructed via the bootstrap test of neighbor-joining algorithm method^28^ based on the 16 S rRNA gene sequences of strain NEAE- H and related organisms.

### Screening of main factors influences melanin production by Plackett–Burman design

Plackett–Burman experimental design is a two factorial design, which identifies the critical environmental and nutritional variables required for elevated melanin production and is very useful for screening the most important factors with respect to their main effects^60^. The total number of experiments to be carried out according to Plackett–Burman is *n* + 1, where *n* is the number of variables^61^. A total of 17 independent (assigned) and two unassigned variables (commonly referred as dummy variables) were screened in Plackett–Burman experimental design. Dummy variables (D_1_ and D_2_) are used to estimate experimental errors in data analysis. Table 2 shows the seventeen different independent variables including incubation period, pH, temperature, agitation speed, medium volume, starch, glycerol, L-tyrosine, potassium nitrate, peptone, protease-peptone, yeast extract, K_2_HPO_4_, ferric ammonium citrate, sodium thiosulfate, MgSO_4_ and NaCl which chosen to be screened by Plackett Burman experiment. Each variable is represented at two levels, high and low denoted by ( + ) and (−), respectively. The experiment was conducted in 20 runs to study the effect of the selected variables on the production of melanin. All trials were performed in duplicate and the average of melanin production was treated as response. Plackett–Burman experimental design is based on the first order model:


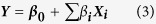


Where, Y is the response or dependent variable (melanin production); it will always be the variable we aim to predict, β_0_ is the model intercept and *β*_*i*_is the linear coefficient, and X_i_ is the level of the independent variable; it is the variable that will help us explain melanin production.

### Face-centered central composite design

The levels and the interaction effects between various significant variables which exerted a positive effect on the melanin production were analyzed and optimized by using face-centered central composite design (FCCD). In this study, the experimental plan consisted of 20 trials and the independent variables were studied at three different levels, low (−1), middle (0) and high ( + 1). All the experiments were done in duplicate and the average of melanin production obtained was taken as the response (Y). The experimental results of FCCD were fitted via the response surface regression procedure using the following second order polynomial equation:


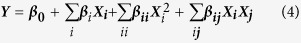


In which Y is the predicted response, β_0_ is the regression coefficients, β_i_ is the linear coefficient, β_ii_ is the quadratic coefficients, β_ij_ is the interaction coefficients and X_i_ is the coded levels of independent variables.

### Statistical analysis

Design Expert^®^ 7.0 software version 7 (Stat-Ease Inc., USA) for Windows was used for the experimental designs and statistical analysis. The statistical software package, STATISTICA software (Version 8.0, StatSoft Inc., Tulsa, USA) was used to plot the three-dimensional surface plots, in order to illustrate the relationship between the responses and the experimental levels of each of the variables utilized in this study.

### Purification of melanin

Melanin was purified by centrifuging the fermentation broth for 15 min at 5000 *g* to remove cells and debris. To precipitate the melanin, the pH of the supernatant was adjusted to 2.0 using 6 M HCl and was allowed to stand for 4 h. The precipitate was then collected by centrifugation at 9000 *g* for 15 min. Melanin pellets were washed with distilled water four times and centrifuged at 9000 *g* for 15 minutes to obtain the purified pigment. Purified pigment was lyophilized and stored at −20 °C until further use.

### UV–visible spectrophotometeric analysis of the purified melanin pigment

The purified melanin powder was first dissolved in 0.5 M NaOH solution and then scanned in a UV–visible spectrophotometer (Optizen pop) at UV, visible and near-infrared wavelengths (200–700 nm). The blank control was 0.5 M NaOH solution^62^.

### Scanning electron microscope

Surface topography of melanin was performed on gold coated samples that had been previously lyophilized using an analytical Scanning Electron Microscope (SEM) (JEOL JSM-6390LV).

### Fourier transform infra red spectroscopy

Fourier transform infrared spectroscopy (FTIR) is most useful for identifying the functional groups and interpretation of structure of unknown compounds. The melanin powder and KBr powder were mixed in an agate mortar and ground for a few seconds to break up the melanin and KBr lumps. The mixed disc was scanned at 4000–400 cm^1^ in an FTIR spectrophotometer (Shimadzu FTIR-8400 S).

### ^1^H NMR

The ^1^H NMR spectrum was obtained by JEOL DELTA2 Nuclear Magnetic Resonance Spectrometer in 5-mm NMR tubes at 25 °C. Operating parameters were: Freq 500.16 MHz, field strength 11.75 T (500 MHz), Resolution 0.57 Hz and acquisition time 1.75 s.

### *In vitro* cytotoxicity and anticancer activities using microculture tetrazolium assay (MTT assay)

Both safety and the anticancer activities of the purified melanin pigment of *Streptomyces glaucescens* strain NEAE-H were measured *in vitro* on both cancerous (skin cancer cell line (HFB4)) and non-cancerous cells (human lung fibroblast (WI-38) and human amnion (WISH)) which obtained from ATCC via holding company for biological products and vaccines (VACSERA), Cairo, Egypt. HFB4 cell line was used to determine the inhibitory effects of melanin pigment on cell growth using standard 3-(4, 5 dimethythiazol-2-yl)-2, 5-diphenyl tetrazolium bromide (MTT) assay^63^. This colorimetric assay is based on the conversion of the yellow tetrazolium bromide (MTT) to a purple formazan derivative by mitochondrial succinate dehydrogenase in viable cells. The cells were cultured in RPMI-1640 medium with 10% fetal bovine serum. Antibiotics added were 100 units/ml penicillin and 100 μg/ml streptomycin at 37 °C in a 5% CO_2_ incubator. The cells were seeded in a 96-well plate at a density of 1.0 × 10^4^cells/well at 37ᵒC for 48 h under 5% CO_2_. After incubation, the cells were treated with different concentration of melanin pigment (1.56, 3.125, 6.25, 12.5, 25, 50 and 100 μg/ml) and incubated for 24 h. After 24 h of drug treatment, 20 μl of MTT solution at 5 mg/ml was added and incubated for 4 h. Dimethyl sulfoxide (DMSO) in volume of 100 μl is added into each well to dissolve the purple formazan formed. The colorimetric assay is measured and recorded at absorbance of 570 nm using a plate reader (EXL 800, USA). The relative cell viability in percentage was calculated as (A570 of treated samples/A570 of untreated sample) X 100. 5-fluorouracil was used as a standard anticancer drug for comparison.

## Conclusion

A melanin producer *Streptomyces glaucescens* strain NEAE-H was isolated from soil sample collected from Al-Taif, Saudi Arabia. The purified melanin exhibited the physical and chemical properties of typical melanin. Maximum melanin yield was obtained using a simple culture process, avoids the use of purified tyrosinase, expensive chemical methods or the cumbersome extraction of this polymer from animal or plant tissues. *Streptomyces glaucescens* strain NEAE-H can be viewed as a promising source of melanin. Melanin pigment produced by *Streptomyces glaucescens* NEAE-H is soluble in water, it is critical for melanin to be water soluble for a better commercial potential in biotechnological applications in the pharmaceutical and cosmetic industries.

## Additional Information

**How to cite this article**: El-Naggar, N. E. and El-Ewasy, S. M. Bioproduction, characterization, anticancer and antioxidant activities of extracellular melanin pigment produced by newly isolated microbial cell factories *Streptomyces glaucescens* NEAE-H. *Sci. Rep.* **7**, 42129; doi: 10.1038/srep42129 (2017).

**Publisher's note:** Springer Nature remains neutral with regard to jurisdictional claims in published maps and institutional affiliations.

## Supplementary Material

Supplementary Information

## Figures and Tables

**Table 1:** Phenotypic properties that separate strain *Streptomyces* sp. strain NEAE-H from related *Streptomyces* species. Data for reference species were taken from Bergey’s Manual of Systematic Bacteriology -volume five the actinobacteria^30^.

Characteristic	*Streptomyces* sp. strain NEAE-H	*Streptomyces glaucescens*	*Streptomyces griseoflavus*	*Streptomyces thinghirensis*	*Streptomyces althioticus*
Aerial mass color on ISP mdeium 2	Green with white margins	Green or blue	Gray	White–grey	Gray
Reverse side of colony on ISP medium 2	Brownish orange	red	Yellow to orange-yellow	Yellow	Blue-violet or red
Production of diffusible pigment	None	None	None	Yellow	Blue-violet or red
Spore chain morphology	Spirals*	Spirals*	Spirals, long	Spirals	Spirals or flexuous, long
Spore surface	Hairy**	Hairy**	Spiny	Smooth	Spiny or warts
Spore shape	Globose to oval			Oval	
**Melanin production on**					
Peptone-yeast extract iron agar	+	+	−	−	−
Tyrosine agar	+	+	−	−	−
Tryptone-yeast extract broth	−	+	−		−
Maximum NaCl tolerance (%, w/v)	5			7	
Coagulation of milk	+			+	
Peptonization of milk	+			+	
Nitrate reduction	–			+	
H_2_S production	−			−	
Gelatin liquification	+			−	
**Utilization of carbon sources (1%,w/v)**					
D(−) fructose	+	+	+	+	+
D(+) xylose	+	+	+	−	+
D(+) galactose	+			+	
D(+) glucose	+	+	+	+	+
L-arabinose	+	+	+	−	+
Ribose	+				
D(+) mannose	+			+	
Sucrose	+	±	±	±	
Maltose	+			±	
Rhamnose	+	+	+	+	+
Raffinose	±	±	±	−	±
Cellulose	+			±	

Abbreviations: +, Positive; −, Negative; ± , Doubtful; Blank cells, no data available. The optimal growth temperature was 30 °C and optimal pH was 7.0. It exhibited no antimicrobial activities against *Staphylococcus aureus, Alternaria solani, Bipolaris oryzae, Sacchromyces cerevisiae, Candida albicans, Bacillus subtilis, Escherichia coli, Pseudomonas aeruginosa, Rhizoctonia solani, Fusarium oxysporum, Aspergillus niger* and *Klebsiella pneumoniae.* Lecithinase activity, α–amylase (starch hydrolysis), protease (degradation of casein), cellulase (growth on cellulose), uricase, gelatinase and asparaginase of strain NEAE-H were produced while chitosanase was not produced.

^*^Mature spore chains are short.

^**^Hairs are coarse, showing some tendency towards spines.

**Table 2:** Twenty-trial Plackett–Burman experimental design for evaluation of nineteen independent variables with coded values along with the melanin production and tyrosinase activity.

Run no.	Coded levels of independent variables																			Melanin production (μg/0.1 ml of medium)		Residuals	Tyrosinase activity (U/ml)
	A	B	C	D	E	F	G	H	J	K	L	M	N	O	P	Q	R	D_1_	D_2_	Actual value	Predicted value		
1	−1	−1	1	1	1	1	−1	1	−1	1	−1	−1	−1	−1	1	1	−1	1	1	5.91	5.991	−0.081	1202.06
2	−1	1	1	−1	1	1	−1	−1	1	1	1	1	−1	1	−1	1	−1	−1	−1	12.57	12.597	−0.027	2284.11
3	1	1	1	1	−1	1	−1	1	−1	−1	−1	−1	1	1	−1	1	1	−1	−1	7.12	7.093	0.027	1666.08
4	1	−1	1	1	−1	−1	1	1	1	1	−1	1	−1	1	−1	−1	−1	−1	1	16.33	16.249	0.081	4148.21
5	1	−1	−1	1	1	1	1	−1	1	−1	1	−1	−1	−1	−1	1	1	−1	1	7.13	7.157	−0.027	1272.06
6	−1	1	−1	1	−1	−1	−1	−1	1	1	−1	1	1	−1	−1	1	1	1	1	6.59	6.509	0.081	1180.06
7	1	−1	1	−1	−1	−1	−1	1	1	−1	1	1	−1	−1	1	1	1	1	−1	14.95	14.869	0.081	1158.06
8	−1	1	1	1	1	−1	1	−1	1	−1	−1	−1	−1	1	1	−1	1	1	−1	5.72	5.747	−0.027	996.05
9	−1	1	−1	−1	−1	−1	1	1	−1	1	1	−1	−1	1	1	1	1	−1	1	16.56	16.533	0.027	4220.21
10	−1	−1	1	1	−1	1	1	−1	−1	1	1	1	1	−1	1	−1	1	−1	−1	7.85	7.823	0.027	1510.08
11	−1	−1	−1	−1	−1	−1	−1	−1	−1	−1	−1	−1	−1	−1	−1	−1	−1	−1	−1	8.97	8.943	0.027	1998.10
12	1	−1	−1	−1	−1	1	1	−1	1	1	−1	−1	1	1	1	1	−1	1	−1	13.82	13.739	0.081	2686.13
13	1	1	−1	−1	1	1	1	1	−1	1	−1	1	−1	−1	−1	−1	1	1	−1	8.35	8.431	−0.081	1680.08
14	1	1	−1	1	−1	1	−1	−1	−1	−1	1	1	−1	1	1	−1	−1	1	1	14.37	14.343	0.027	3052.15
15	−1	1	1	−1	−1	1	1	1	1	−1	1	−1	1	−1	−1	−1	−1	1	1	6.92	6.839	0.081	1838.09
16	−1	−1	−1	−1	1	1	−1	1	1	−1	−1	1	1	1	1	−1	1	−1	1	12.53	12.557	−0.027	2762.14
17	1	−1	1	−1	1	−1	−1	−1	−1	1	1	−1	1	1	−1	−1	1	1	1	17.96	18.041	−0.081	5454.27
18	−1	−1	−1	1	1	−1	1	1	−1	−1	1	1	1	1	−1	1	−1	1	−1	14.09	14.171	−0.081	2934.15
19	1	1	−1	1	1	−1	−1	1	1	1	1	−1	1	−1	1	−1	−1	−1	−1	16.65	16.677	−0.027	1608.08
20	1	1	1	−1	1	−1	1	−1	−1	−1	−1	1	1	−1	1	1	−1	−1	1	11.49	11.571	−0.081	1866.09
Level	days		^ο^C	rpm	ml	g/L	g/L	g/L	g/L	g/L	g/L	g/L	g/L	g/L	g/L	g/L	g/L						
−1	2	7	30	100	50	0	0	1	0	7	2	0.5	0.5	0.1	0.01	0	0						
1	5	8.5	37	200	100	5	5	3	2	15	4	1	1	0.4	0.08	0.5	0.5						

A (incubation period); B (pH); C (temperature); D (agitation speed); E (medium volume, ml/250 ml flask); F (starch); G (glycerol); H (L-tyrosine); J (potassium nitrate); K (peptone); L (protease-peptone); M (yeast extract); N (K_2_HPO_4_); O (ferric ammonium citrate); P (sodium thiosulfate); Q (MgSO_4_) and R (NaCl).

**Table 3:** Regression statistics and analysis of variance (ANOVA) for the experimental results of Plackett-Burman design used for melanin production by *Streptomyces glaucescens* strain NEAE-H.

Source	*SS*	*MS*	*F-*value	*p*-value Prob > *F*	Confidence Level (%)
Model	328.37	19.32	529.93	0.0019*	99.810
Incubation period (A)	46.39	46.39	1272.72	0.0008*	99.920
pH (B)	8.71	8.71	239.01	0.0042*	99.580
Temperature (C)	7.49	7.49	205.51	0.0048*	99.520
Agitation speed (D)	25.00	25.00	685.83	0.0015*	99.850
Starch (F)	53.60	53.60	1470.38	0.0007*	99.930
Glycerol (G)	4.38	4.38	120.18	0.0082*	99.180
L-tyrosine (H)	8.37	8.37	229.69	0.0043*	99.570
Peptone (K)	18.62	18.62	510.96	0.0020*	99.800
Protease-peptone (L)	51.91	51.91	1424.04	0.0007*	99.930
Yeast extract (M)	7.64	7.64	209.56	0.0047*	99.530
K_2_HPO_4_ (N)	0.87	0.87	23.74	0.0396*	96.040
Ferric ammonium citrate (O)	65.74	65.74	1803.55	0.0006*	99.940
Sodium thiosulfate (P)	9.55	9.55	261.99	0.0038*	99.620
MgSO_4_ (Q)	1.47	1.47	40.30	0.0239*	97.610
(R) NaCl	13.38	13.38	367.15	0.0027*	99.730
Residual	0.07	0.04			
Cor Total	328.44				
Std. Dev.	0.191		R-Squared		0.9998
Mean	11.294		Adj R-Squared		0.9979
C.V.%	1.690		Pred R-Squared		0.9778
PRESS	7.29		Adeq Precision		67.8771

^*^Significant values, *SS* - sum of squares, *MS*- mean square, *F*: Fishers’s function, *P*: Level of significance, PRESS -the predicted residual sum of squares, CV %-the coefficient of variation%.

**Table 4:** Face-centered central composite design representing the melanin production by *Streptomyces glau*c*escens* strain NEAE-H as influenced by incubation period (X_1_), protease-peptone (X_2_) and ferric ammonium citrate (X_3_) along with the predicted melanin production and residuals and the levels of variables with actual factor levels corresponding to coded factor levels.

Std	Run	Variables			Melanin production (μg/0.1 ml of medium)		Residuals	Tyrosinase activity (U/ml)
		X_1_	X_2_	X_3_	Experimental	Predicted		
17	1	0	0	0	31.650	30.592	1.058	6089.10
14	2	0	0	1	13.993	15.539	−1.546	3981.80
20	3	0	0	0	31.650	30.592	1.058	6089.10
10	4	1	0	0	24.059	24.942	−0.883	4087.40
7	5	−1	1	1	4.658	4.564	0.093	2503.33
4	6	1	1	−1	4.597	4.835	−0.238	2496.12
5	7	−1	−1	1	7.362	6.331	1.031	3331.37
1	8	−1	−1	−1	8.067	6.596	1.471	3367.37
13	9	0	0	−1	13.003	14.630	−1.628	3784.99
19	10	0	0	0	31.650	30.592	1.058	6089.10
3	11	−1	1	−1	3.529	3.834	−0.305	2251.31
12	12	0	1	0	26.838	25.711	1.127	4497.82
18	13	0	0	0	31.650	30.592	1.058	6089.10
9	14	−1	0	0	23.620	25.911	−2.290	4051.40
15	15	0	0	0	31.650	30.592	1.058	6089.10
8	16	1	1	1	6.240	6.917	−0.677	3062.55
2	17	1	−1	−1	3.006	2.306	0.700	2174.51
6	18	1	−1	1	4.491	3.392	1.099	2462.52
11	19	0	−1	0	21.029	25.330	−4.301	4008.20
16	20	0	0	0	31.650	30.592	1.058	6089.10
**Level**	**Incubation period (day)**				**Protease-peptone (g/L)**	**Ferric ammonium citrate (g/L)**		
**−1**	4				3	0.3		
**0**	6				5	0.5		
**1**	8				7	0.7		

**Table 5:** Regression statistics, analysis of variance (ANOVA) for FCCD results used for optimizing melanin production by *Streptomyces glaucescens* strain NEAE-H.

Source	Coefficient estimate	Sum of Squares	*df*	Mean Square	*F-*value	*P-*value *P*rob > *F*
Model		2594.424	9	288.269	66.903	< 0.0001*
X_1_	−0.484	2.345	1	2.345	0.544	0.4776
X_2_	0.191	0.364	1	0.364	0.084	0.7774
X_3_	0.454	2.063	1	2.063	0.479	0.5047
X_1_ X_2_	1.323	13.998	1	13.998	3.249	0.1016
X_1_ X^3^	0.338	0.914	1	0.914	0.212	0.6550
X_2_ X_3_	0.249	0.496	1	0.496	0.115	0.7415
X_1_^2^	−5.166	73.378	1	73.378	17.030	0.0021*
X_2_^2^	−5.072	70.742	1	70.742	16.418	0.0023*
X_3_^2^	−15.508	661.349	1	661.349	153.490	<0.0001*
Residual		43.088	10	4.309		
Lack of Fit		43.088	5	8.618		
Pure Error		0.000	5	0.000		
Cor Total		2637.511	19			
Std. Dev.	2.076	R-Squared	0.9837			
Mean	17.720	Adj R-Squared	0.9690			
C.V.%	11.715	Pred R-Squared	0.9025			
PRESS	257.139	Adeq Precision	19.2714			

^*^Significant values, *df*: Degree of freedom, *F*: Fishers’s function, *P*: Level of significance, C.V: Coefficient of variation, intercept coefficient estimate: 30.592.

**Table 6:** Cytotoxicity and anticancer activities of various concentrations of the purified melanin pigment of *Streptomyces glaucescens* strain NEAE-H on both cancerous and non-cancerous cells. 5-fluorouracil was used as a standard anticancer drug for comparison.

Concentration (μg/ml)	Cytotoxicity (%)		
	Skin cancer cell line (HFB4)	Human lung fibroblast (WI-38)	Human amnion (WISH)
**5-fluorouracil**			
1.56	5.9	12.7	24.8
3.125	21.8	35	37.1
6.25	45.1	49.4	54.9
12.5	61.7	66.9	72.4
25	75.6	78.6	81.7
50	83.8	85.8	90.3
100	92.2	92.5	94.2
Cytotoxicity IC_50_ (μg/ml)	8.85 ± 0.52	6.68 ± 0.57	5.07 ± 0.38
**Melanin pigment**			
1.56	0	0	0
3.125	7.7	0	0
6.25	28.2	9.8	3.5
12.5	49.4	32.9	25.9
25	66	43.7	37.8
50	70.9	57.3	51
100	81.3	68.1	64.4
Cytotoxicity IC_50_ (μg/ml)	16.34 ± 1.31	37.05 ± 2.40	48.07 ± 2.76

IC_50_ (μg/ml): 1–10 (very strong). 11–20 (strong). 21–50 (moderate). 51–100 (weak) and above 100 (non-cytotoxic).
