# Correction: Bioproduction, characterization, anticancer and antioxidant activities of extracellular melanin pigment produced by newly isolated microbial cell factories *Streptomyces glaucescens* NEAE-H

**DOI:** 10.1038/s41598-025-34495-1

**Published:** 2026-01-21

**Authors:** Noura El-Ahmady El-Naggar, Sara M. El-Ewasy

**Affiliations:** Department of Bioprocess Development, Genetic Engineering and Biotechnology Research Institute, City for Scientific Research and Technological Applications, Alexandria, Egypt

Correction to: *Scientific Reports* 10.1038/srep42129, published online 14 February 2017

This Article contains errors. This article contains typographical errors, where all instances of HFB4 should read A-431.

Therefore, under the Results and Discussion section, under the subheading “*In vitro* anticancer activity,” the sentences:

The anti-proliferative activity of the purified melanin pigment of *Streptomyces glaucescens* strain NEAE-H was assayed for its anticancer activity in vitro against skin cancer cell line (HFB4) using MTT assay.

MTT assay revealed that melanin pigment produced by *Streptomyces glaucescens* strain NEAE-H showed potent cytotoxic activity against HFB4 skin cancer cell line.

The potent cytotoxic activity of melanin against HFB4 skin cancer cell line and low cytotoxicity of against normal non-cancerous cells shows that melanin pigment can be used as potential natural anticancer.

should read:

The anti-proliferative activity of the purified melanin pigment of *Streptomyces glaucescens* strain NEAE-H was assayed for its anticancer activity in vitro against skin cancer cell line (A-431) using MTT assay.

MTT assay revealed that melanin pigment produced by *Streptomyces glaucescens* strain NEAE-H showed potent cytotoxic activity against A-431 skin cancer cell line.

The potent cytotoxic activity of melanin against A-431 skin cancer cell line and low cytotoxicity of against normal non-cancerous cells shows that melanin pigment can be used as potential natural anticancer.

In Table 6 the column heading “Skin cancer cell line (HFB4)” should read “Skin cancer cell line (A-431)”.

and under the Methods section, under the subheading “*In vitro* cytotoxicity and anticancer activities using microculture tetrazolium assay (MTT assay),” the sentence:

Both safety and the anticancer activities of the purified melanin pigment of *Streptomyces glaucescens* strain NEAE-H were measured *in vitro* on both cancerous (skin cancer cell line (HFB4)) and non-cancerous cells (human lung fibroblast (WI-38) and human amnion (WISH)) which obtained from ATCC via holding company for biological products and vaccines (VACSERA), Cairo, Egypt. HFB4 cell line was used to determine the inhibitory effects of melanin pigment on cell growth using standard 3-(4, 5 dimethythiazol-2-yl)-2, 5-diphenyl tetrazolium bromide (MTT) assay^63^.

should read:

Both safety and the anticancer activities of the purified melanin pigment of *Streptomyces glaucescens* strain NEAE-H were measured in vitro on both cancerous (skin cancer cell line (A-431)) and non-cancerous cells (human lung fibroblast (WI-38) and human amnion (WISH)) which obtained from ATCC via holding company for biological products and vaccines (VACSERA), Cairo, Egypt. A-431 cell line was used to determine the inhibitory effects of melanin pigment on cell growth using standard 3-(4, 5 dimethythiazol-2-yl)-2, 5-diphenyl tetrazolium bromide (MTT) assay^63^.

