# Supplementary material for: Bioproduction, characterization, anticancer and antioxidant activities of extracellular melanin pigment produced by newly isolated microbial cell factories Streptomyces glaucescens NEAE-H
Source: Sci Rep. 2017 Feb 14;7:42129. doi: 10.1038/srep42129 (PMC5307326; doi:10.1038/srep42129)
Supplement: Supplementary Information [file srep42129-s1.doc]

**Bioproduction, characterization, cytotoxicity and anticancer activities of extracellular melanin pigment produced by newly isolated microbial cell factories** ***Streptomyces glaucescens* NEAE-H**

**Noura El-Ahmady El-Naggar*, Sara M. El-Ewasy**

Department of Bioprocess Development, Genetic Engineering and Biotechnology Research Institute, City for Scientific Research and Technological Applications, Alexandria, Egypt

**Running title:** **Bioproduction of extracellular melanin pigments**

*To whom correspondence should be addressed.

**Dr. Noura El-Ahmady Ali El-Naggar**

**Address:**

Bioprocess Development Department,

Genetic Engineering and Biotechnology Research Institute,

City of Scientific Research and Technological Applications,

New Borg El- Arab City, 21934, Alexandria, Egypt

**Tel:** (002)01003738444

**Fax:** (002)03 4593423

**E-mail:** nouraelahmady@yahoo.com

**Supplementary Table S1. Culture characteristics of the *Streptomyces* sp. strain NEAE-H.**

| **Medium** | **Color of** | | | **Growth** |
| --- | --- | --- | --- | --- |
|  | **Aerial**  **mycelium** | **Substrate mycelium** | **Diffusible pigment** |  |
| ISP medium 1  (Tryptone-yeast extract agar) | Green | Yellowish brown | Non-pigmented | Excellent |
| ISP medium 2  (Yeast extract -malt extract agar) | Green with white margins | Brownish orange | Non-pigmented | Excellent |
| ISP medium 3  (Oatmeal agar) | Dark green | Yellowish green | Yellow | Excellent |
| ISP medium 4  (Inorganic salt-starch agar) | Green | Brown | Faint brown | Excellent |
| ISP medium 5  (Glycerol asparagines agar) | Green | Brown | Faint brown | Very good |
| ISP medium 6  (Peptone-yeast extract iron agar) | Grayish white | Faint brown | Brown | Very good |
| ISP medium 7  (Tyrosine agar) | Grayish green | Brown | Brown | Very good |

The substrate mycelium pigment was not pH sensitive when tested with 0.05 N NaOH or 0.05 N HCl.

**Supplementary Table S2.** Regression coefficients, estimated effect and % of contribution for melanin production by *Streptomyces glaucescens* strain NEAE-H using Plackett–Burman design.

| **Term** | **Coefficient** | **Effect** | **% Contribution** |
| --- | --- | --- | --- |
| Intercept | 11.294 |  |  |
| A | 1.523 | 3.05 | 11.160 |
| B | -0.66 | -1.32 | 4.830 |
| C | -0.612 | -1.22 | 4.464 |
| D | -1.118 | -2.24 | 8.196 |
| F | -1.637 | -3.27 | 11.965 |
| G | -0.468 | -0.94 | 3.439 |
| H | 0.647 | 1.29 | 4.720 |
| K | 0.965 | 1.93 | 7.062 |
| L | 1.611 | 3.22 | 11.782 |
| M | 0.618 | 1.24 | 4.537 |
| N | 0.208 | 0.42 | 1.537 |
| O | 1.813 | 3.63 | 13.282 |
| P | 0.691 | 1.38 | 5.049 |
| Q | -0.271 | -0.54 | 1.976 |
| R | -0.818 | -1.64 | 6.001 |

**Supplementary Table S3.** Fit summary for FCCD results

| **Sequential Model Sum of Squares** | | | | | | | | |
| --- | --- | --- | --- | --- | --- | --- | --- | --- |
| **Source** | **Sum of Squares** | ***df*** | **Mean Square** | | ***F-*value** | | ***P-*value**  ***P*rob >*F*** | |
| Linear vs Mean | 4.772 | 3 | 1.591 | | 0.0097 | | 0.9986 | |
| 2FI vs Linear | 15.407 | 3 | 5.136 | | 0.0255 | | 0.9942 | |
| Quadratic vs 2FI | 2574.245 | 3 | 858.082 | | 199.1483 | | < 0.0001* | |
| Residual | 23.081 | 6 | 3.847 | |  | |  | |
| **Lack of Fit Tests** | | | | | | | | |
| **Source** | **Sum of Squares** | ***df*** | **Mean Square** | | ***F-*value** | | ***P-*value**  ***P*rob >*F*** | |
| Linear | 2632.739 | 11 | 239.340 | |  | |  | |
| 2FI | 2617.332 | 8 | 327.167 | |  | |  | |
| Quadratic | 43.088 | 5 | 8.618 | |  | |  | |
| Pure Error | 0 | 5 | 0 | |  | |  | |
| **Model Summary Statistics** | | | | | | | | |
| **Source** | **Standard deviation** | **R-Squared** | | **Adjusted R-Squared** | | **Predicted R-Squared** | | **PRESS** |
| Linear | 12.828 | 0.0018 | | -0.1854 | | -0.7310 | | 4565.607 |
| 2FI | 14.189 | 0.0077 | | -0.4504 | | -5.8601 | | 18093.72 |
| Quadratic | 2.076 | 0.9837 | | 0.9690 | | 0.9025 | | 257.1388 |
| * Significant values,  *df* : degree of freedom, PRESS: sum of squares of prediction error, Two factors interaction: 2FI | | | | | | | | |


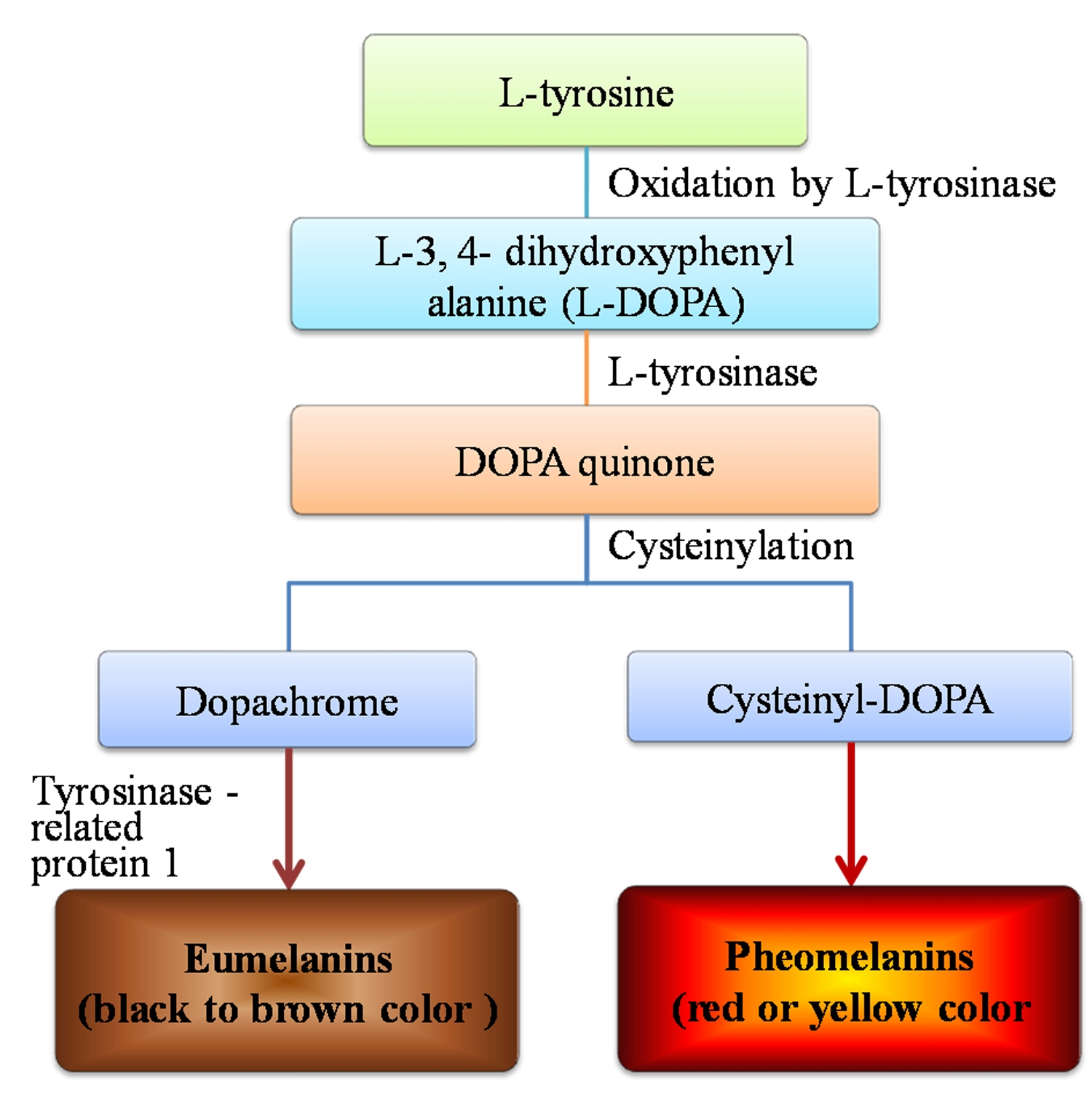
**Supplementary Figure S1**. Schematic representation of melanin biosynthetic pathway

**
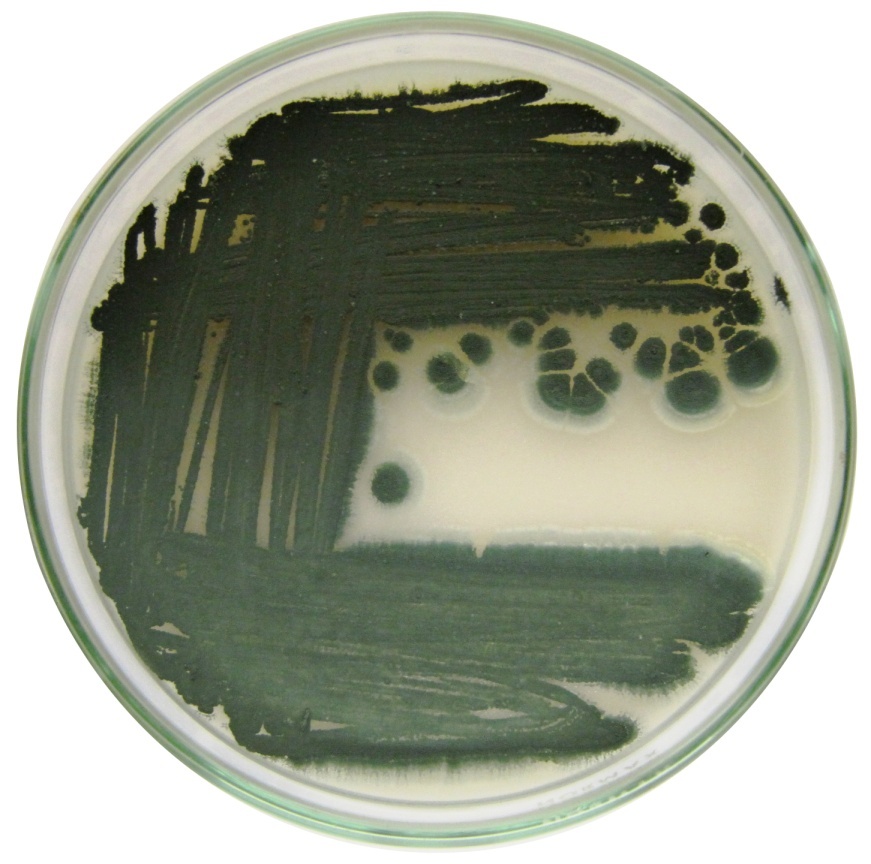
**

**Supplementary Figure S2**. Green color of the mature sporulating aerial mycelium of *Streptomyces* sp. NEAE-H grown on starch-nitrate agar.

**Experimental section:**

**Morphology and cultural characteristics**

The morphology of the spore chain and the spore surface ornamentation of strain NEAE-H were examined on starch nitrate agar medium after 14 days of incubation at 30°C. The gold-coated dehydrated specimen can be examined at different magnifications with Analytical Scanning Electron Microscope Jeol JSM-6360 LA operating at 20 Kv at the Central Laboratory, City for Scientific Research and Technology Applications, Alexandria, Egypt. Aerial spore-mass colour, substrate mycelial pigmentation and the production of diffusible pigments were observed on, yeast extract-malt extract agar (ISP medium 2), oatmeal agar (ISP medium 3), inorganic salt starch agar (ISP medium 4), glycerol- asparagine agar (ISP medium 5) peptone-yeast extract iron agar (ISP medium 6), tyrosine agar (ISP medium 7) as described by Shirling and Gottlieb [1]; all plates were incubated at 30°C for 14 days.

**Physiological characteristics**

Carbon source utilization was tested on plates containing ISP basal medium 9 and melanoid pigment production was examined on ISP medium 1, 6, 7 following the methods of Shirling and Gottlieb^1^. Growth in the presence of sodium chloride was determined according to Tresner *et al*.^2^. Degradation of casein was tested following the method of Gordon *et al*.^3^ and reduction of nitrates to nitrites was examined^4^. Liquefaction of gelatin was evaluated by using the method of Waksman^5^. The ability to coagulate or to peptonize milk was determined as described by Cowan and Steel^6^. Lecithinase activity was conducted on egg–yolk medium according to the method of Nitsch and Kützner^7^ and the capacity to decompose cellulose was tested following the method of Ariffin *et al*.^8^. The ability of strain to produce α-amylase (starch degradation) was also determined; the isolate was streaked onto starch nitrate medium plate containing 2% soluble starch and incubated at 30°C for 7 days. After incubation, the plate is flooded with Gram's iodine solution and zone of clearance was observed^9^. The ability of the organism to inhibit the growth of four bacterial (*Pseudomonas aeruginosa*, *Staphylococcus aureus*, *Escherichia coli*, or *Klebsiella*), and five fungal strains (*Rhizoctonia solani*, *Alternaria solani*, *Bipolaris oryzae*, *Fusarium oxysporum*, *Fusarium solani*) was determined using the plug agar method. The strain was lawn-cultured by dense streaking on starch nitrate medium plates and incubated at 30 ºC for seven days. Nine mm agar discs were prepared using sterile cork borer from well grown culture and placed on fresh lawn culture of the test organisms. The plates were then kept at 4 ºC for overnight for the diffusion of the antimicrobial metabolites, and then incubated 30 ºC. The zones of inhibition were determined after 24 h for bacteria and 48 h for fungi.

**ABTS^+^ radical scavenging (anti-oxidant) activity assay**

The purified melanin pigment of *Streptomyces glaucescens* strain NEAE-H was tested for anti-oxidant activity. For melanin pigment, 2 ml of 2, 2′-Azino-bis (3-ethylbenzthiazoline-6-sulfonic acid (ABTS) solution (60 µM) was added to 3 M MnO_2_ solution (25 mg/ml) all prepared in phosphate buffer (pH 7, 0.1 M). The mixture was shaken, centrifuged, filtered, and the absorbance (A_control_) of the resulting green-blue solution (ABTS radical solution) was adjusted at ca. 0.5 at λ 734 nm. Then, 50 µl of (2 mM) solution of the pigment in spectroscopic grade MeOH/ phosphate buffer (1:1) was added. The absorbance (A_test_) was measured and the reduction in color intensity was expressed as % inhibition. The % inhibition is calculated from the following equation^10^:

$\text{\% Inhibition = }\frac{\text{A}_{\text{Control}}\text{ -A}_{\text{Test}}}{\text{A}_{\text{Control}}}\text{ }\times\text{ 100 }$

Ascorbic acid (vitamin C) was used as standard anti-oxidant (positive control). Negative control sample was run with MeOH/phosphate buffer (1:1) instead of melanin pigment.

**Assay for erythrocyte hemolysis.**

Blood was obtained from rats by cardiac puncture and collected in heparinized tubes. Erythrocytes were separated from plasma and the buffy coat and washed three times with 10 volumes of 0.15 M NaCl. During the last washing, the erythrocytes were centrifuged at 2500 *g* for 10 min to obtain a constantly packed cell preparation. Erythrocyte hemolysis was mediated by peroxyl radicals in this assay system^11^. A 10% suspension of erythrocytes in pH 7.4 phosphate-buffered saline (PBS) was added to the same volume of 200 mM 2,20-azobis (2-amidinopropane) dihydrochloride (AAPH) solution (in PBS) containing samples to be tested at different concentrations. The reaction mixture was shaken gently while being incubated at 37 ᵒC for~h. The reaction mixture was then removed, diluted with eight volumes of PBS and centrifuged at 2500 *g* for 10 min. The absorbance A of the supernatant was read at 540 nm. Similarly, the reaction mixture was treated with eight volumes of distilled water to achieve complete hemolysis, and the absorbance B of the supernatant obtained after centrifugation was measured at 540 nm. The percentage hemolysis was calculated by equation:

$$\text{\% Hemolysis = 1-}\frac{\text{A}}{\text{B}}\text{ }\times\text{ 100 }$$

The data were expressed as mean standard deviation. L-ascrobic was used as a positive control.

**References**

1. Shirling, E. & Gottlieb, D. (1966) Methods for characterization of *Streptomyces* species1. *Int J Syst Evol Microbiol* **16**, 313-340.

2. Tresner, H., Hayes, J. A. & Backus, E. Differential tolerance of streptomycetes to sodium chloride as a taxonomic aid. *Appl Microbiol* **16**, 1134-1136 (1968).

3. Gordon, R. E., Barnett, D. A., Handerhan, J. E. & Pang, C. H. N. *Nocardia coeliaca*, *Nocardia autotrophica*, and the *nocardin* strain. *Int J Syst Evol Microbiol* **24**, 54-63 (1974).

4. Williams, S., Goodfellow, M., Alderson, G., Wellington, E., Sneath, P. & Sackin, M. Numerical classification of *Streptomyces* and related genera. *Microbiol* **129**, 1743-1813 (1983).

5. Waksman, S. A. The Actinomycetes. Vol. II. Classification, identification and descriptions of genera and species. The Actinomycetes Vol II Classification, identification and descriptions of genera and species (1961). Williams & Wilkins Co, Baltimore.

6. Cowan, S. T. Cowan & Steel’s Manual for the identiﬁcation of medical bacteria. Cambridge University, London (1974). ISBN: 0521203996 Record Number: 19742282078

7. Nitsch, B. & Kützner, H. Egg-yolk agar as a diagnostic medium for streptomycetes. *Experientia*  **25**, 220-221(1969).

8. Ariffin, H., Abdullah, N., Umi Kalsom, M., Shirai, Y. & Hassan, M. Production and characterization of cellulase by *Bacillus pumilus* EB3. *Int J Eng Technol* **3**, 47-53 (2006).

9. Mishra, S. & Behera, N. Amylase activity of a starch degrading bacteria isolated from soil receiving kitchen wastes. *African J Biotechnol* **7(18)**, 3326-3331(2008).

10. Lissi, E., Modak, B., Torres, R., Escobar, J. & Urzua, A. Total antioxidant potential of resinous exudates from *Heliotropium* species, and a comparison of ABTS and DPPH methods. *Free Radic Res* **30**, 471-477(1999).

11. Morimoto, Y. et al. Protective effects of some neutral amino acids against hypotonic hemolysis. *Biol Pharm Bull* 18**(10)**, 1417-1422(1995).
